# Author Correction: Preferential inhibition of adaptive immune system dynamics by glucocorticoids in patients after acute surgical trauma

**DOI:** 10.1038/s41467-020-18410-y

**Published:** 2020-09-03

**Authors:** Edward A. Ganio, Natalie Stanley, Viktoria Lindberg-Larsen, Jakob Einhaus, Amy S. Tsai, Franck Verdonk, Anthony Culos, Sajjad Ghaemi, Kristen K. Rumer, Ina A. Stelzer, Dyani Gaudilliere, Eileen Tsai, Ramin Fallahzadeh, Benjamin Choisy, Henrik Kehlet, Nima Aghaeepour, Martin S. Angst, Brice Gaudilliere

**Affiliations:** 1 Department of Anesthesiology, Perioperative and Pain Medicine, School of Medicine, Stanford University, Stanford, CA USA; 2 The Lundbeck Foundation Center for Fast-track Hip and Knee replacement, Copenhagen, Denmark; 3 Digital Technologies Research Centre, National Research Council Canada, Toronto, ON Canada; 4 Division of Plastic and Reconstructive Surgery, Department of Surgery, School of Medicine, Stanford University, Stanford, CA USA; 5 Section of Surgical Pathophysiology 7621, Rigshospitalet, Blegdamsvej 9, DK-2100 Copenhagen, Denmark

**Keywords:** Bioinformatics, Acute inflammation, Signal transduction, Trauma

Correction to: *Nature Communications* 10.1038/s41467-020-17565-y, published online 27 July 2020.

The original version of this article contained an error in the spelling of the author Sajjad Ghaemi which was incorrectly given as Sajjad Gahemi. This has now been corrected in both the PDF and HTML versions of the article.

