# Supplementary material for: Preferential inhibition of adaptive immune system dynamics by glucocorticoids in patients after acute surgical trauma
Source: Nat Commun. 2020 Jul 27;11:3737. doi: 10.1038/s41467-020-17565-y (PMC7385146; doi:10.1038/s41467-020-17565-y)
Supplement: Supplementary file 1 — Supplementary Information [file 41467_2020_17565_MOESM1_ESM.pdf]

Supplementary Information: Preferential inhibition of adaptive immune system dynamics by glucocorticoids in patients after acute surgical trauma

Ganio et al.

Supplementary Figure 1. CONSORT chart

Supplementary Figure 2. Gating Strategy

Supplementary Figure 3. Overview of cluster plots for frequency and all measured intracellular signaling markers

Supplementary Figure 4-15. Cell frequency and intracellular signaling in manually gated cell subsets.

Supplementary Figure 16. Alteration of intracellular pSTAT5 responses by MP

Supplementary Table 1. Mass cytometry panel

Supplementary Table 2. Two-sided Wilcoxon rank sum p-values

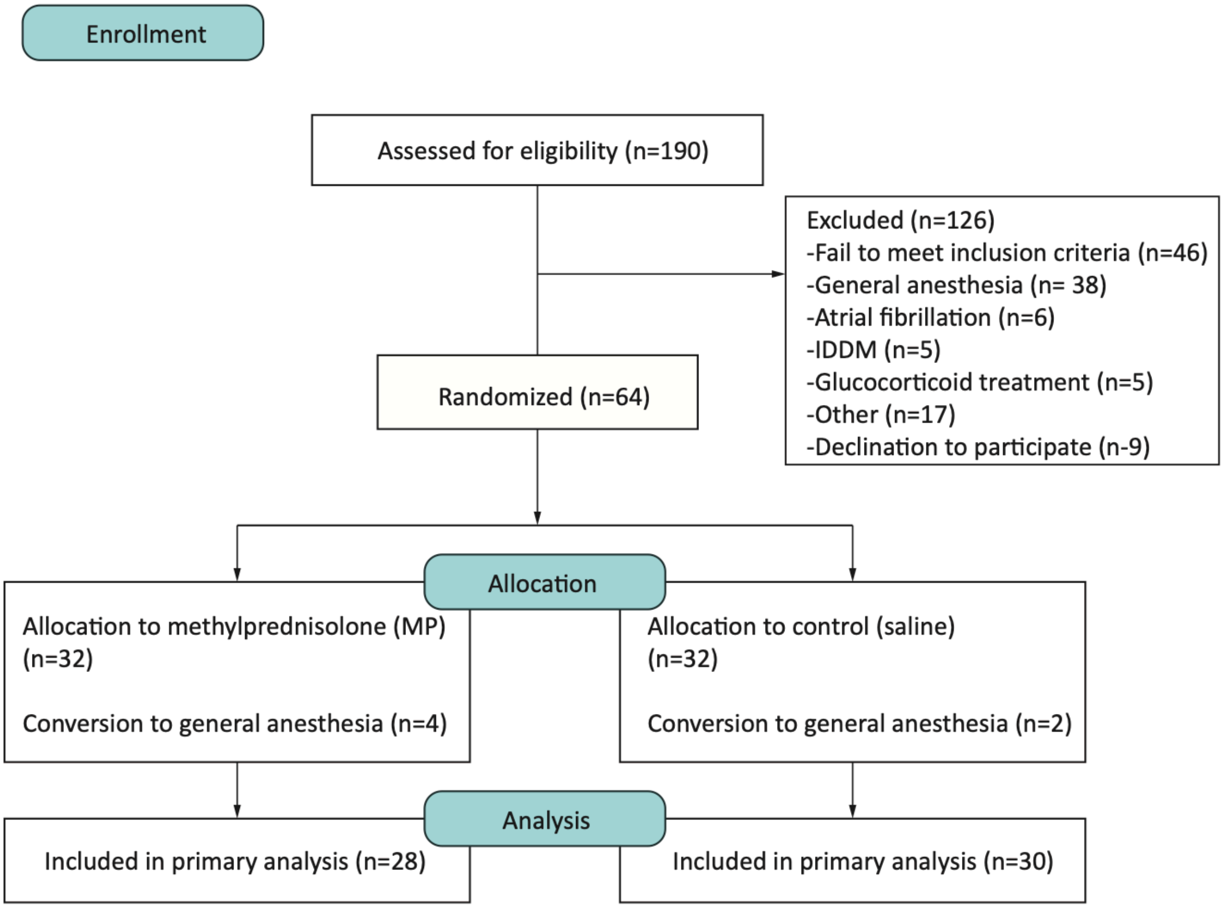

Supplementary Figure 1. CONSORT map.

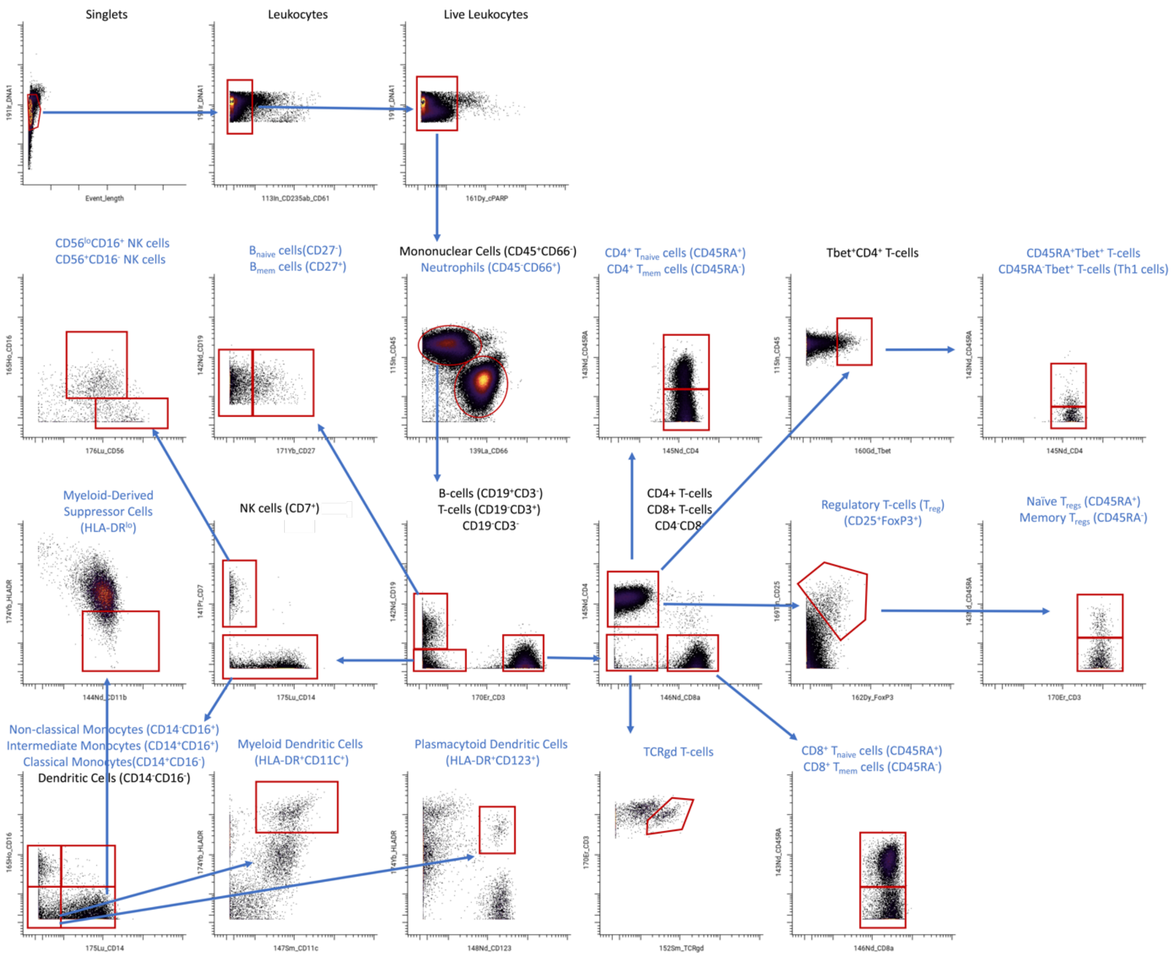

Supplementary Figure 2. Gating Strategy. Two-dimensional dot plots depicted for a representative sample. Gating was performed using Cell Engine (https://cellengine.com). Live singlet leukocytes were derived from DNA$^+$CD235ab$^-$CD61$^-$ cPARP$^-$ events, which are split into mononuclear cells (CD45$^+$CD66$^-$) and neutrophils (CD45$^+$CD66$^+$). Initially, adaptive cell populations are gated from the mononuclear cells (CD19$^+$CD3$^-$ B cells and CD19$^-$CD3$^+$ T cells). The CD19$^-$CD3$^-$ population is further split into CD7$^+$ NK cells (CD56^{lo}CD16$^+$ and CD56^{hi}CD16$^-$, respectively) and CD14$^+$CD16$^-$ classical monocytes, CD14$^+$CD16$^+$ intermediate monocytes, and CD14$^-$CD16$^+$ non-classical monocytes. Dendritic cell populations are defined as CD14$^-$CD16$^-$HLA-DR$^+$, and subdivided into CD11c$^+$ mDC, and CD123$^+$ pDC. A subpopulation of myeloid-derived suppressor cells (HLA-DR^{lo}) is derived from classical monocytes. B cells are split into naïve (CD27$^-$) and memory (CD27$^+$) cells. T cells are divided into TCRgd$^+$, CD4$^+$, and CD8$^+$ subpopulations. Among the CD4$^+$ T cells, T helper 1 (Tbet$^+$), regulatory T (FoxP3$^+$CD25$^+$), naïve (CD45RA$^+$), and memory (CD45RA$^-$) cells are identified. Among the CD8$^+$ T cells, naïve (CD45RA$^+$), and memory (CD45RA$^-$) cells are identified. Cell types included in analysis are labeled in blue.

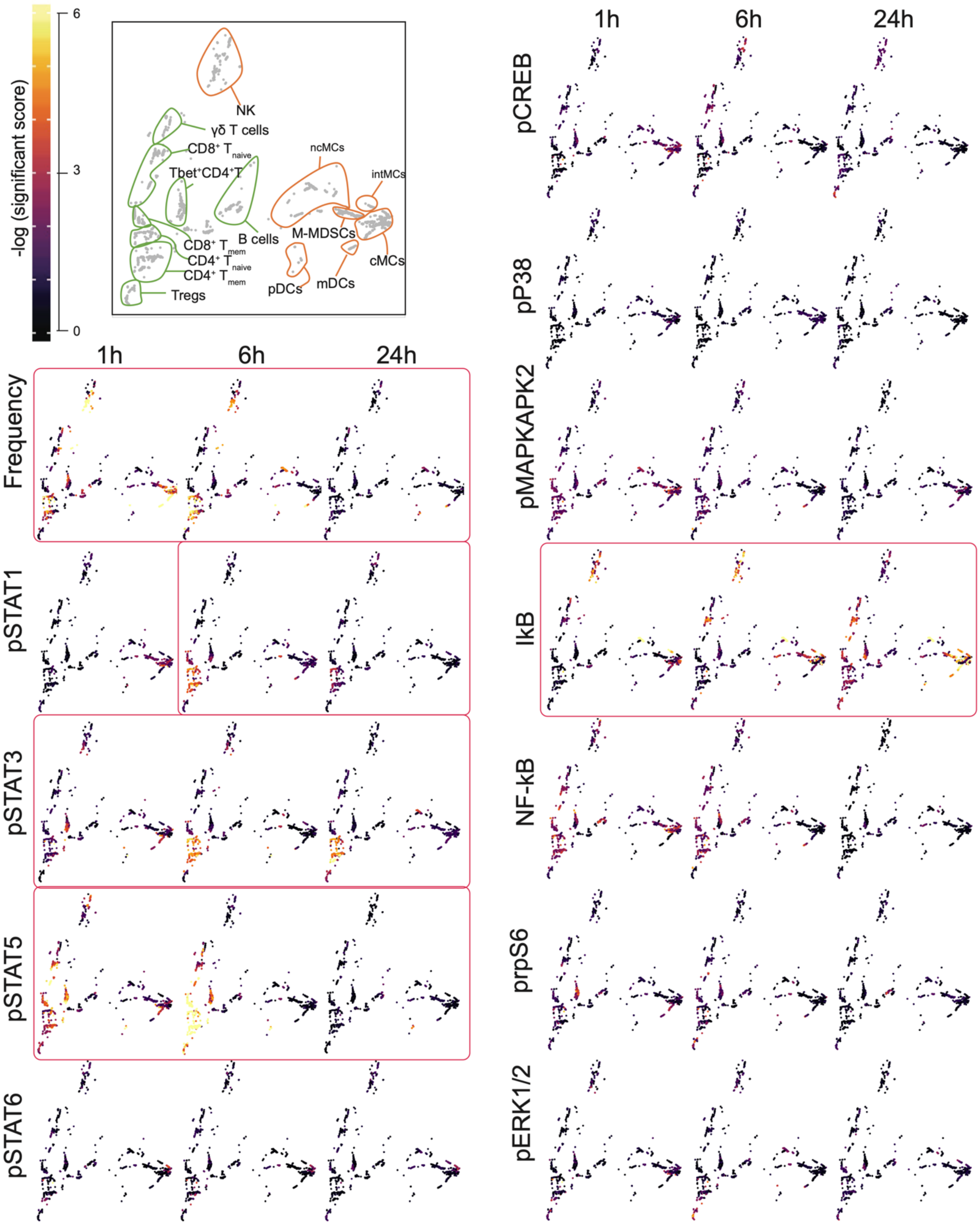

Supplementary Figure 3. Overview of cluster plots for frequency and all measured intracellular signaling markers. Significant time points and markers boxed in magenta. Univariate p-values (two-sided Wilcoxon Rank Sum Test) were computed for each cluster at each time point to quantify the difference in functional marker expression between samples in the control and MP groups.

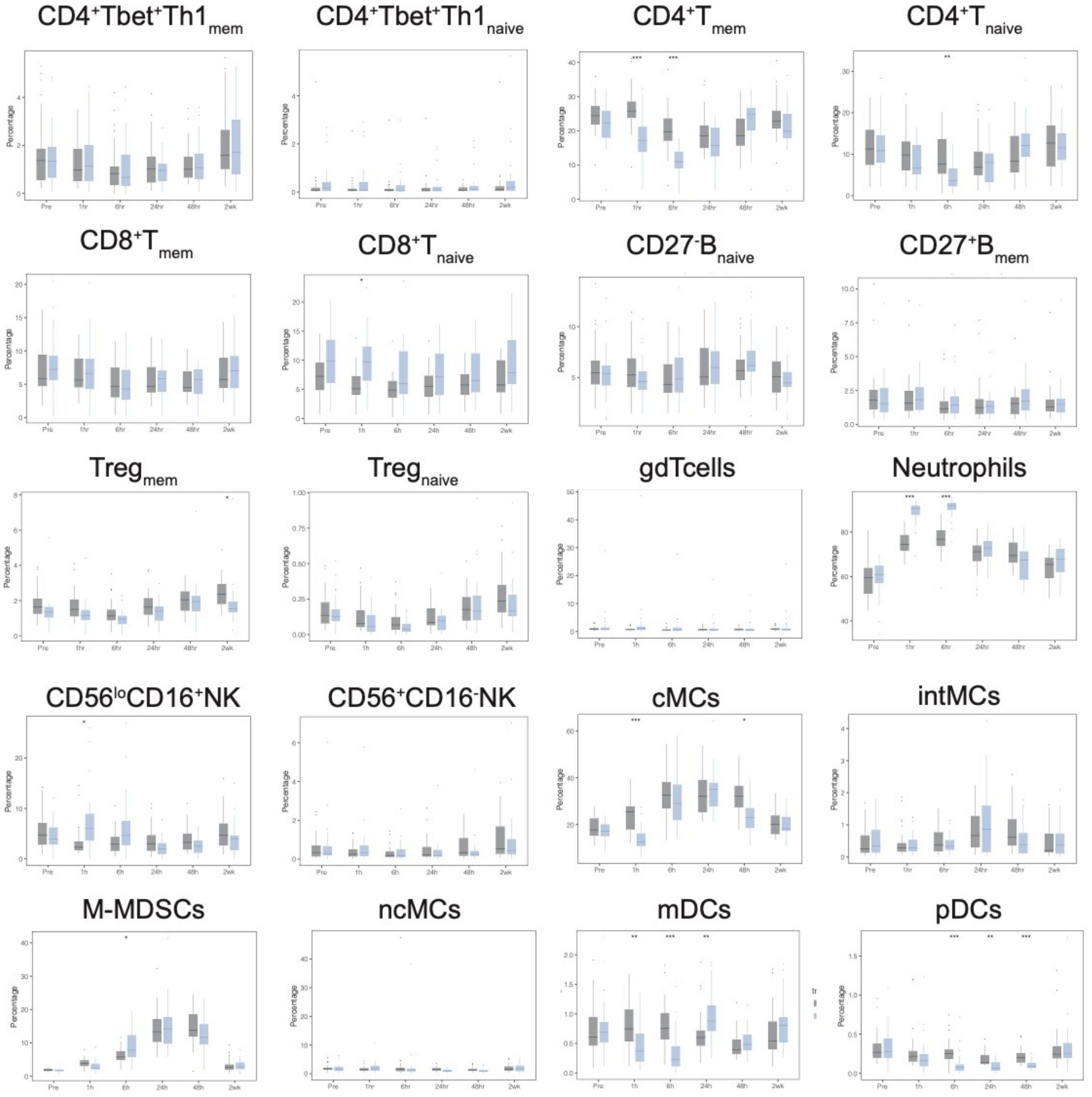

Supplementary Figure 4. Cell frequency in manually gated cell subsets. Boxplots depict cell frequency (y-axis) as a percentage of live cells (neutrophils) or CD45+ cells (all mononuclear cell types) in manually gated cell subsets before surgery (Pre) and 1h, 6h, 24h, 48h, and 2 weeks after surgery (x-axis) in control (n=30 patients receiving saline; gray) and MP (n=30 patients receiving MP; blue). All boxplots show median values, interquartile range, and whiskers of 1.5 times interquartile range. (Two-sided Wilcoxon rank-sum test. * = $p<0.01$, ** = $p<0.001$, *** = $p<0.0001$, p-values in **Supplementary Table 2**).

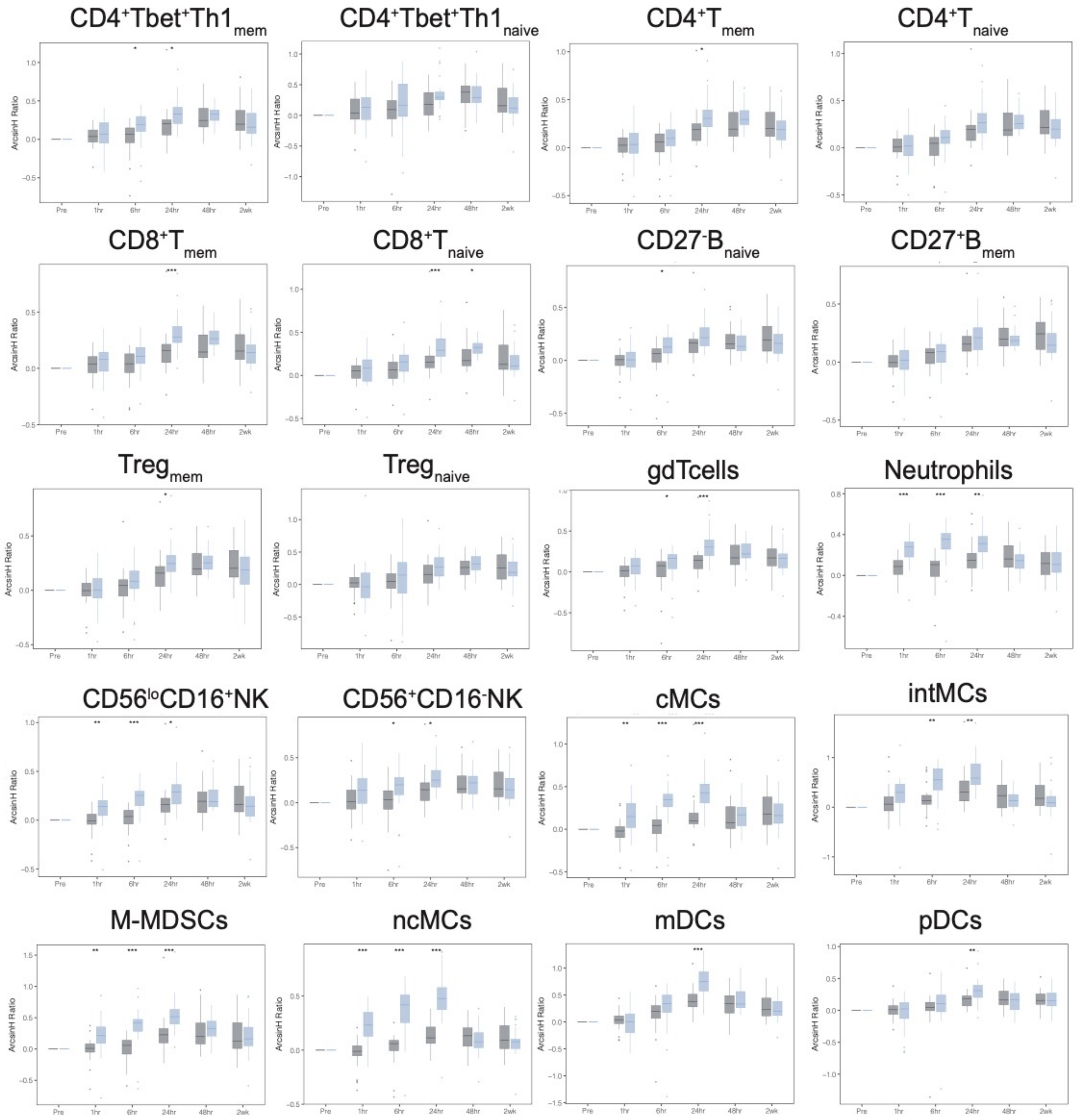

Supplementary Figure 5. Intracellular signaling response for total IκBα in manually gated cell subsets. Boxplots depict ArcSinh ratio over the endogenous signal response (y-axis) in 20 manually gated cell subsets before surgery (Pre) and 1h, 6h, 24h, 48h, and 2 weeks after surgery (x-axis) in control (n=30 patients; gray) and MP (n=28 patients; blue). All boxplots show median values, interquartile range, and whiskers of 1.5 times interquartile range. (Two-sided Wilcoxon rank-sum test. * = p<0.01, ** = p<0.001, *** = p<0.0001, p-values in **Supplementary Table 2**).

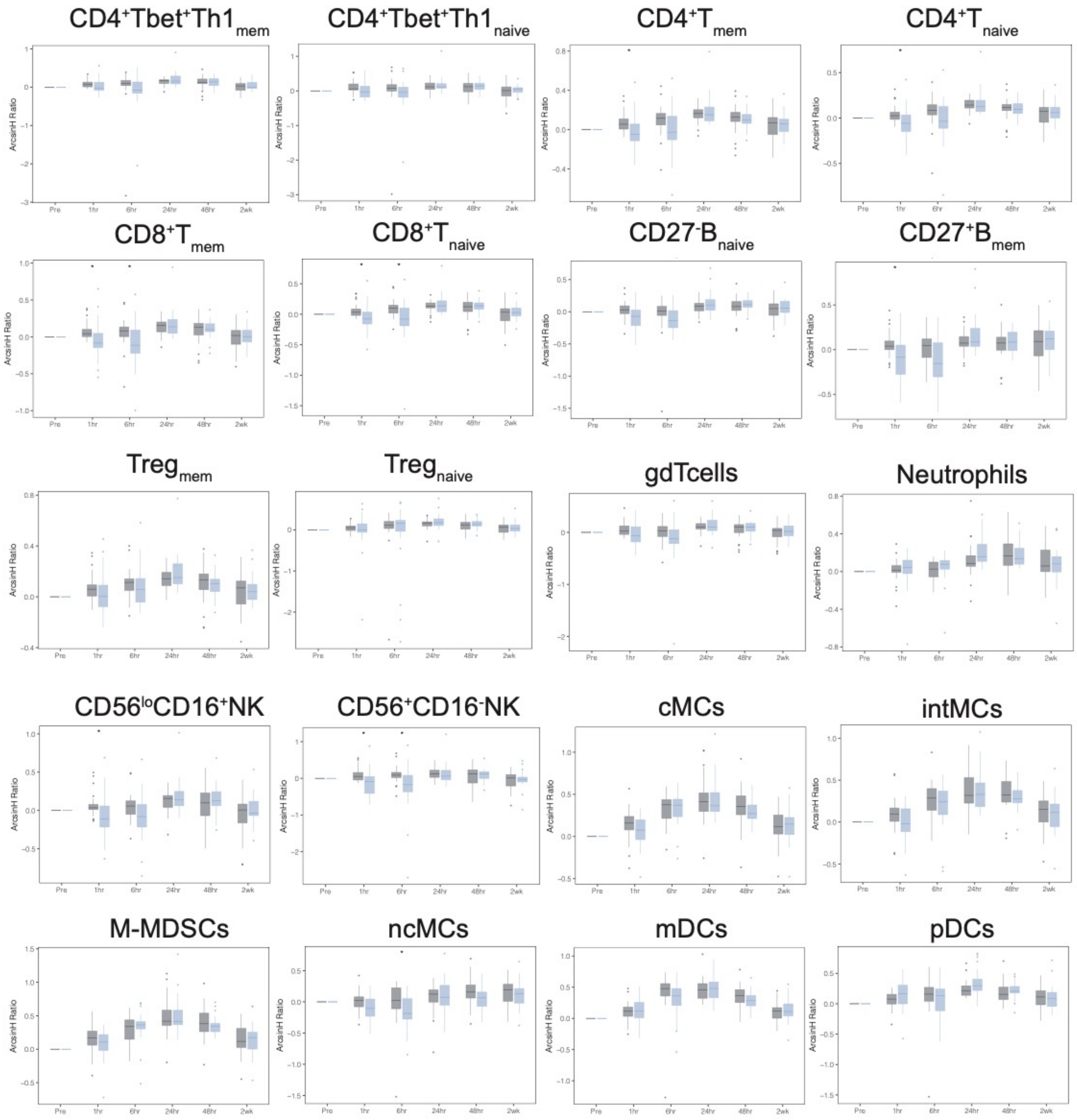

Supplementary Figure 6. Intracellular signaling response for phospho-S529 NF-κB p65 subunit (RelA) in manually gated cell subsets. Boxplots depict ArcSinh ratio over the endogenous signal response (y-axis) in 20 manually gated cell subsets before surgery (Pre) and 1h, 6h, 24h, 48h, and 2 weeks after surgery (x-axis) in control (n=30 patients; gray) and MP (n=28 patients; blue). All boxplots show median values, interquartile range, and whiskers of 1.5 times interquartile range. (Two-sided Wilcoxon rank-sum test. * = p<0.01, ** = p<0.001, *** = p<0.0001, p-values in **Supplementary Table 2**).

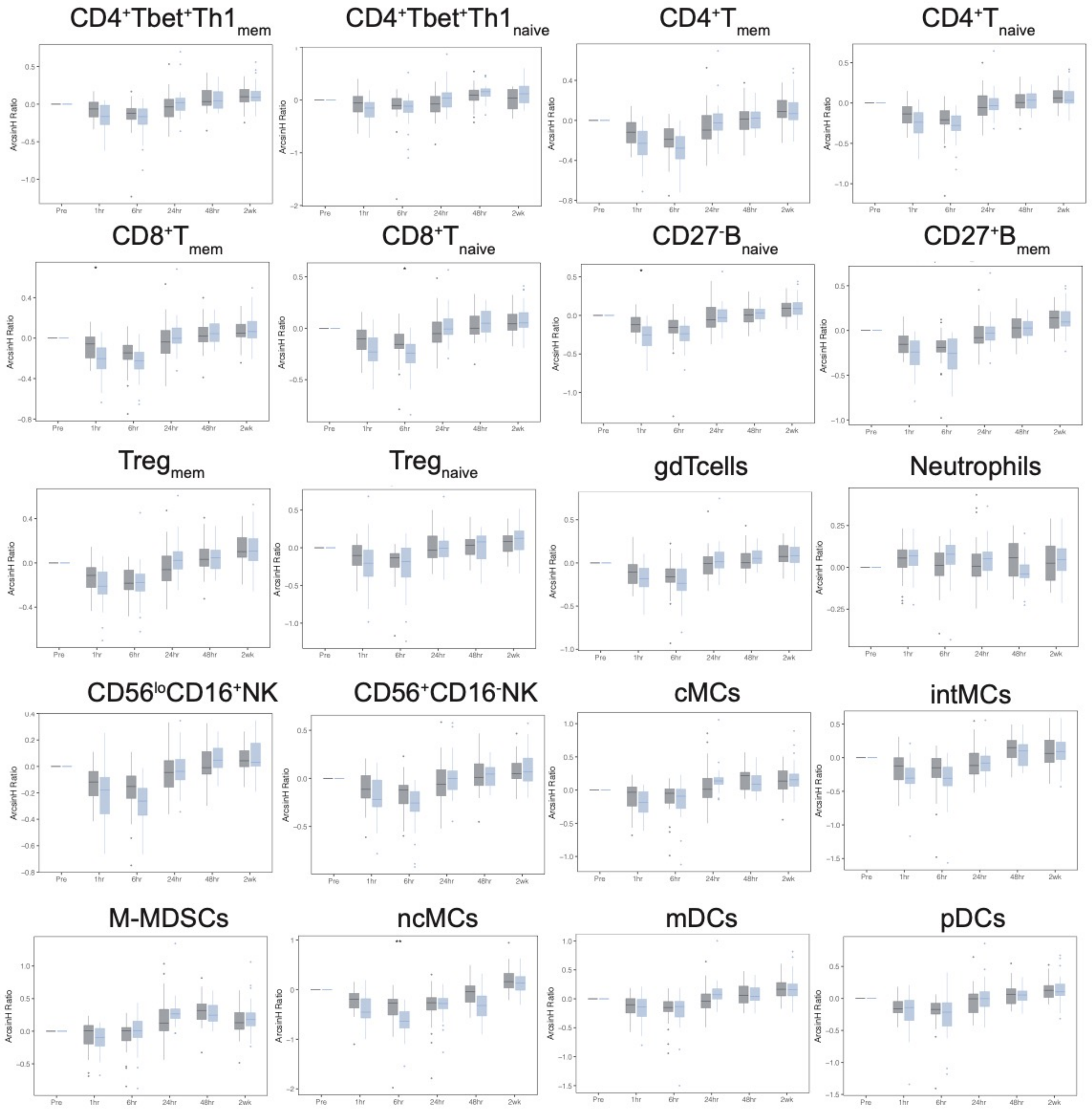

Supplementary Figure 7. Intracellular signaling response for phospho-T334 MAPKAPK2 in manually gated cell subsets. Boxplots depict ArcSinh ratio over the endogenous signal response (y-axis) in 20 manually gated cell subsets before surgery (Pre) and 1h, 6h, 24h, 48h, and 2 weeks after surgery (x-axis) in control (n=30 patients; gray) and MP (n=28 patients; blue). All boxplots show median values, interquartile range, and whiskers of 1.5 times interquartile range. (Two-sided Wilcoxon rank-sum test. * = p<0.01, ** = p<0.001, *** = p<0.0001, p-values in **Supplementary Table 2**).

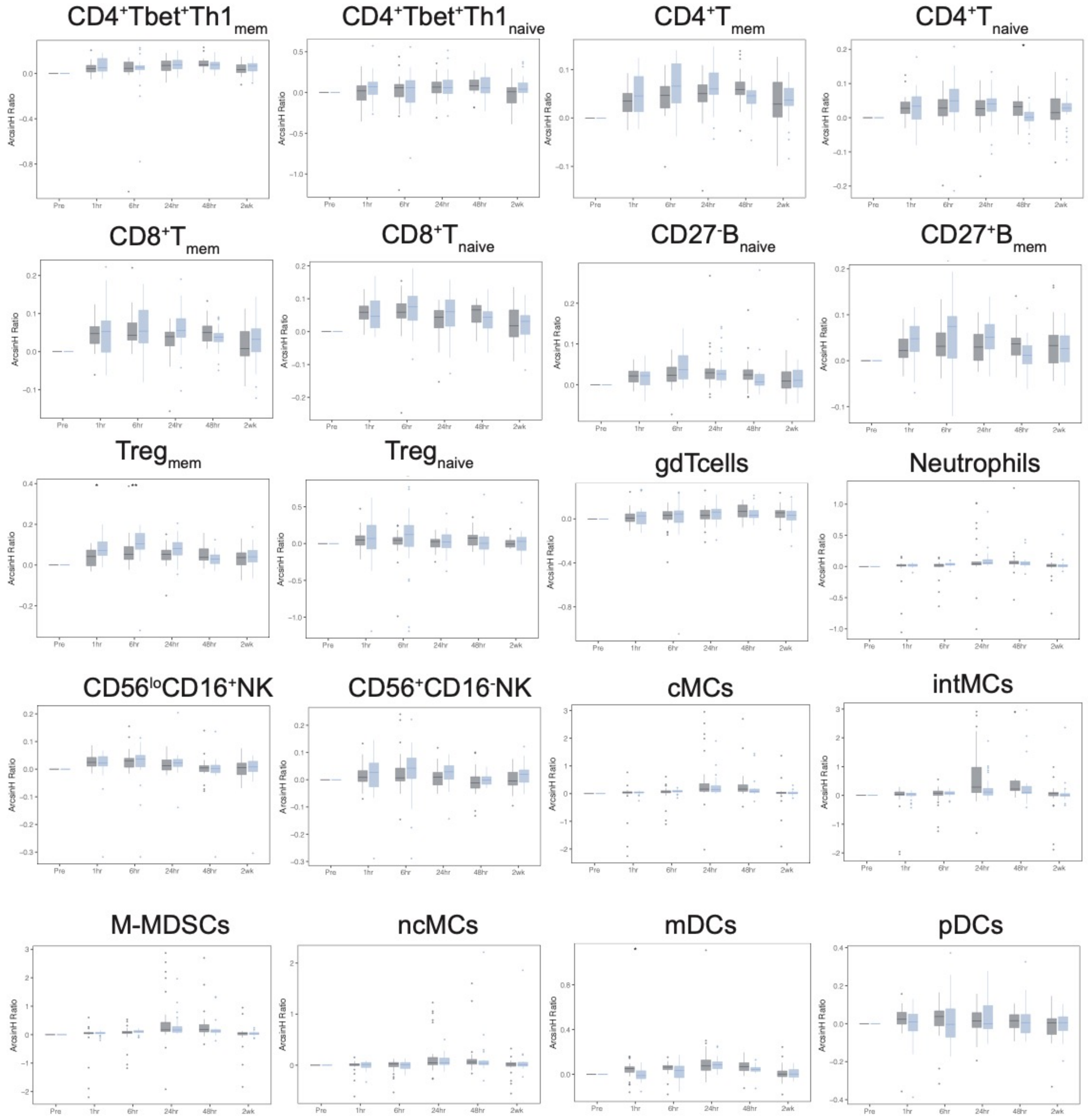

Supplementary Figure 8. Intracellular signaling response for phospho-T202/Y204 ERK1/2 in manually gated cell subsets. Boxplots depict ArcSinh ratio over the endogenous signal response (y-axis) in 20 manually gated cell subsets before surgery (Pre) and 1h, 6h, 24h, 48h, and 2 weeks after surgery (x-axis) in control (n=30 patients; gray) and MP (n=28 patients; blue). All boxplots show median values, interquartile range, and whiskers of 1.5 times interquartile range. (Two-sided Wilcoxon rank-sum test. * = p<0.01, ** = p<0.001, *** = p<0.0001, p-values in **Supplementary Table 2**).

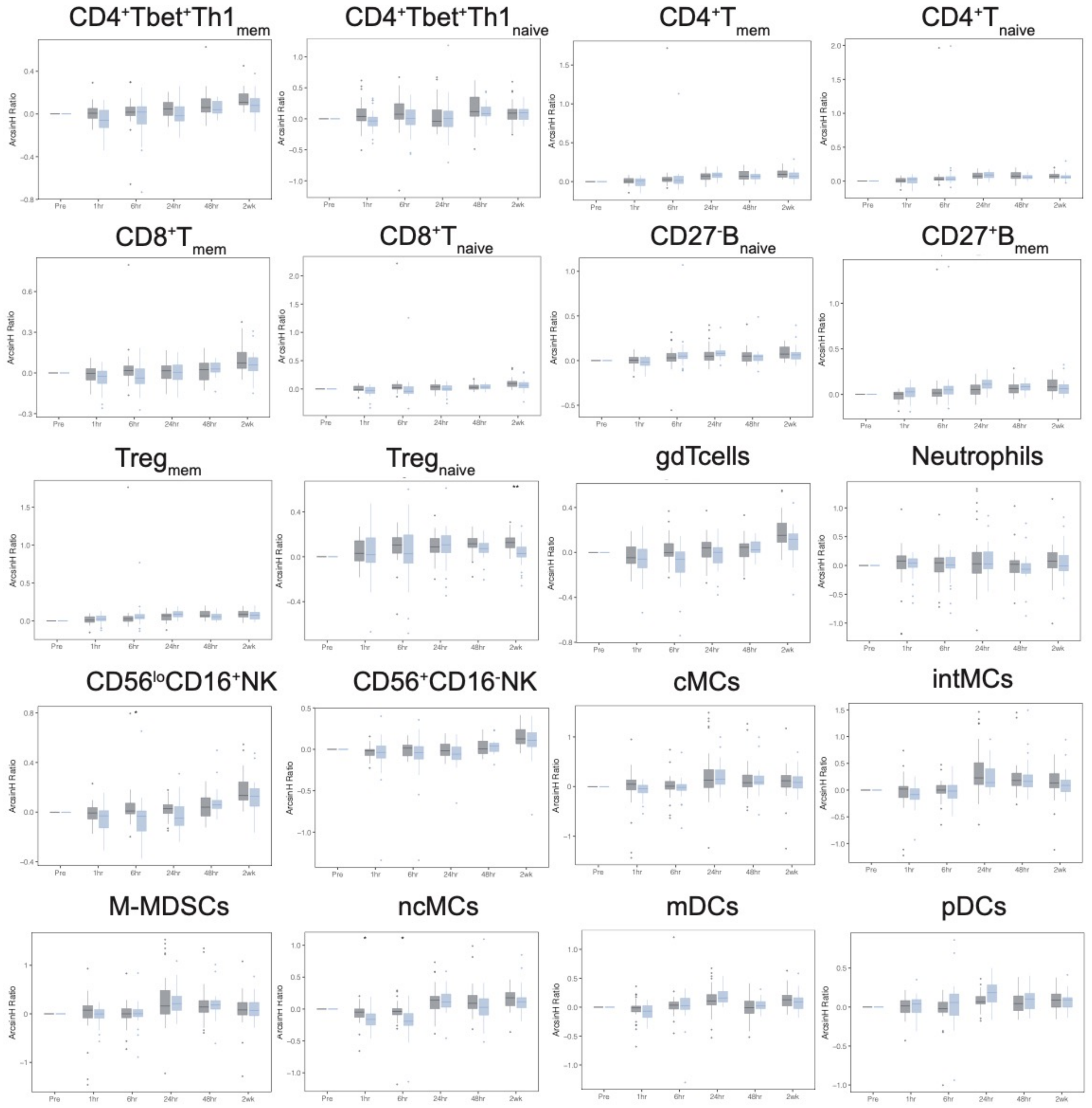

Supplementary Figure 9. Intracellular signaling response for phospho-S133 CREB in manually gated cell subsets. Boxplots depict ArcSinh ratio over the endogenous signal response (y-axis) in 20 manually gated cell subsets before surgery (Pre) and 1h, 6h, 24h, 48h, and 2 weeks after surgery (x-axis) in control (n=30 patients; gray) and MP (n=28 patients; blue). All boxplots show median values, interquartile range, and whiskers of 1.5 times interquartile range. ((Two-sided Wilcoxon rank-sum test. * = p<0.01, ** = p<0.001, *** = p<0.0001, p-values in **Supplementary Table 2**).

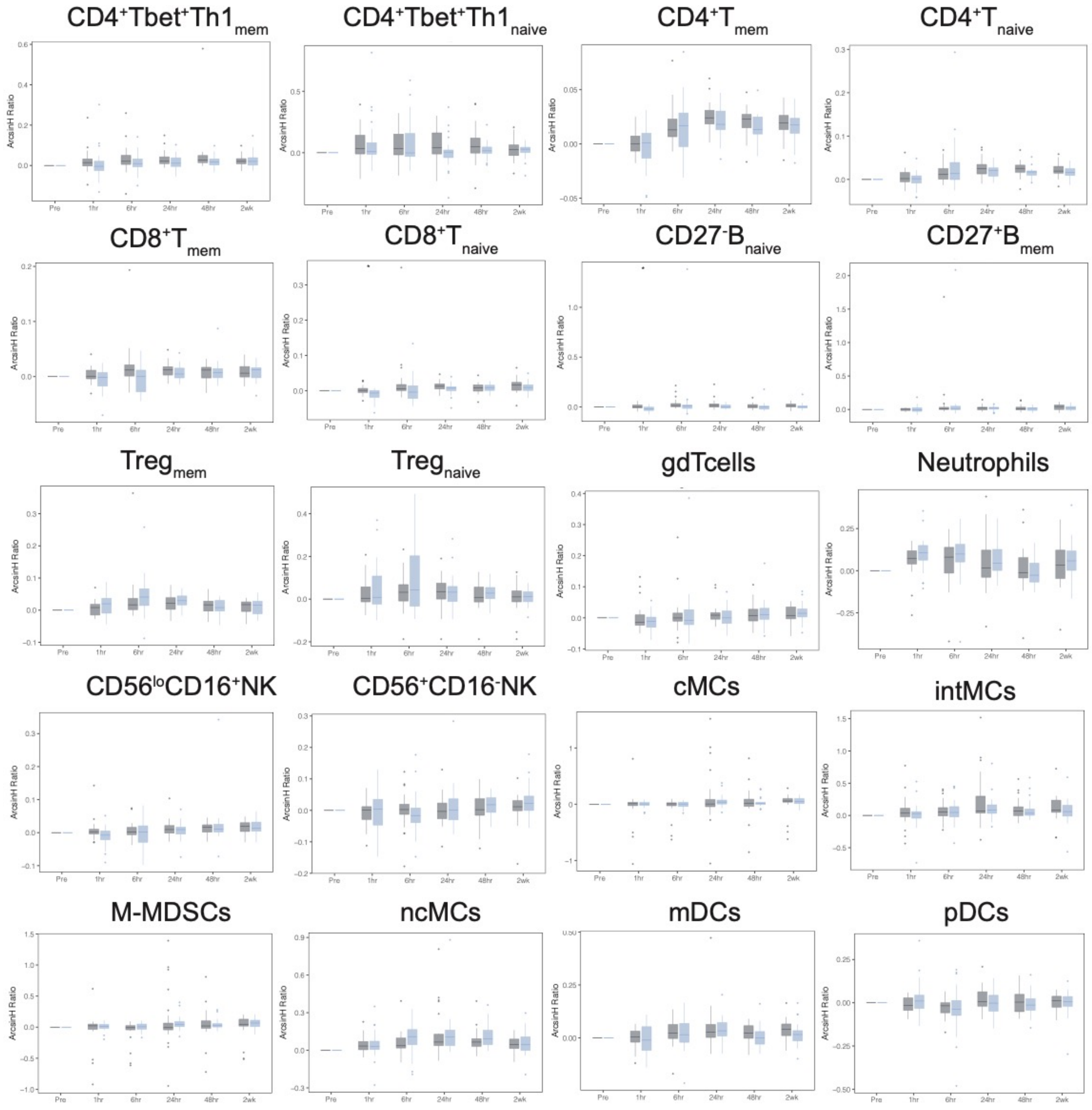

Supplementary Figure 10. Intracellular signaling response for phospho-S235/S236 rpS6 in manually gated cell subsets. Boxplots depict ArcSinh ratio over the endogenous signal response (y-axis) in 20 manually gated cell subsets before surgery (Pre) and 1h, 6h, 24h, 48h, and 2 weeks after surgery (x-axis) in control (n=30 patients; gray) and MP (n=28 patients; blue). All boxplots show median values, interquartile range, and whiskers of 1.5 times interquartile range. (Two-sided Wilcoxon rank-sum test. * = p<0.01, ** = p<0.001, *** = p<0.0001, p-values in **Supplementary Table 2**).

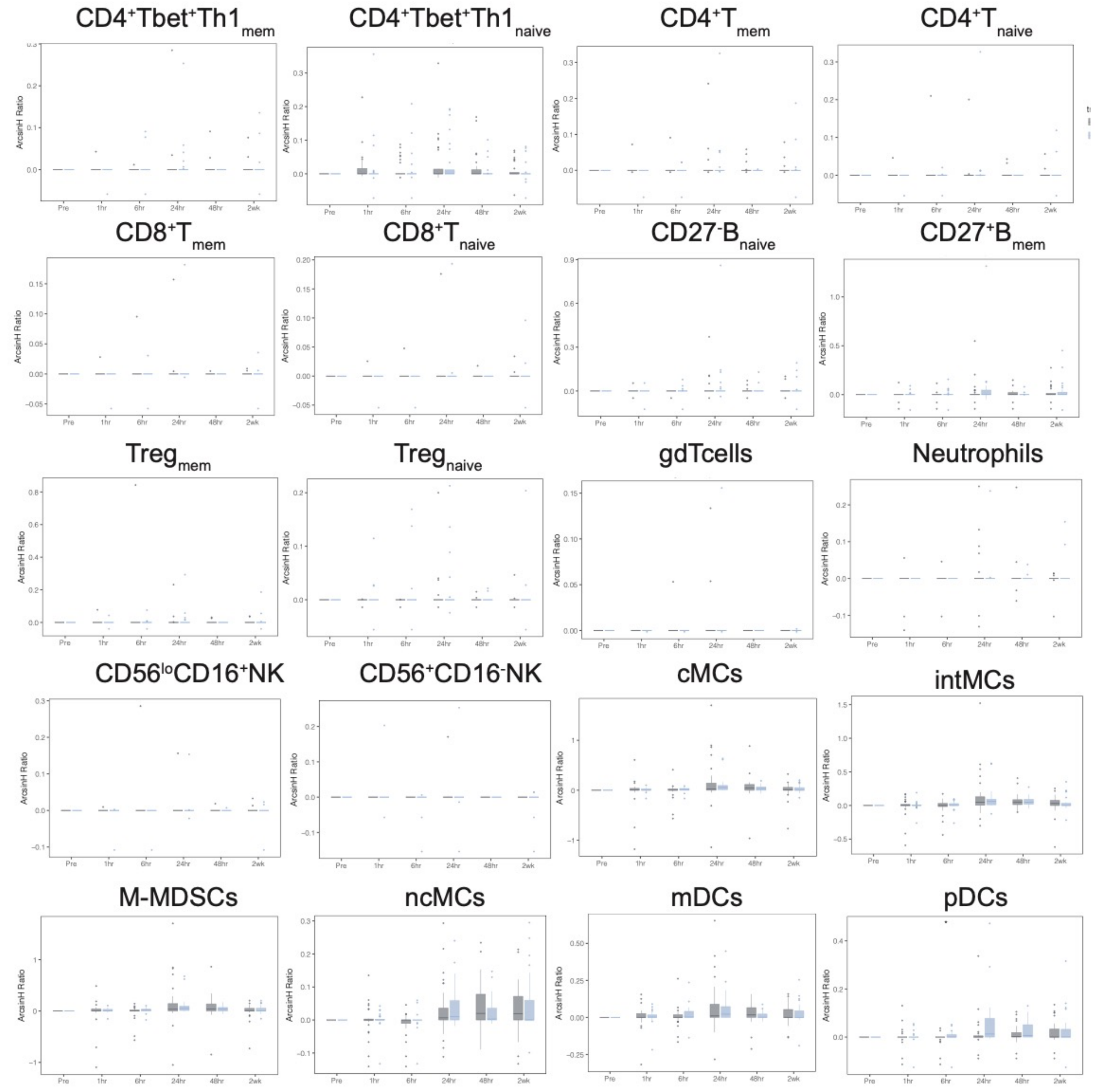

Supplementary Figure 11. Intracellular signaling response for phospho-T180/Y182 P38 in manually gated cell subsets. Boxplots depict ArcSinh ratio over the endogenous signal response (y-axis) in 20 manually gated cell subsets before surgery (Pre) and 1h, 6h, 24h, 48h, and 2 weeks after surgery (x-axis) in control (n=30 patients; gray) and MP (n=28 patients; blue). All boxplots show median values, interquartile range, and whiskers of 1.5 times interquartile range. (Two-sided Wilcoxon rank-sum test. * = p<0.01, ** = p<0.001, *** = p<0.0001, p-values in **Supplementary Table 2**).

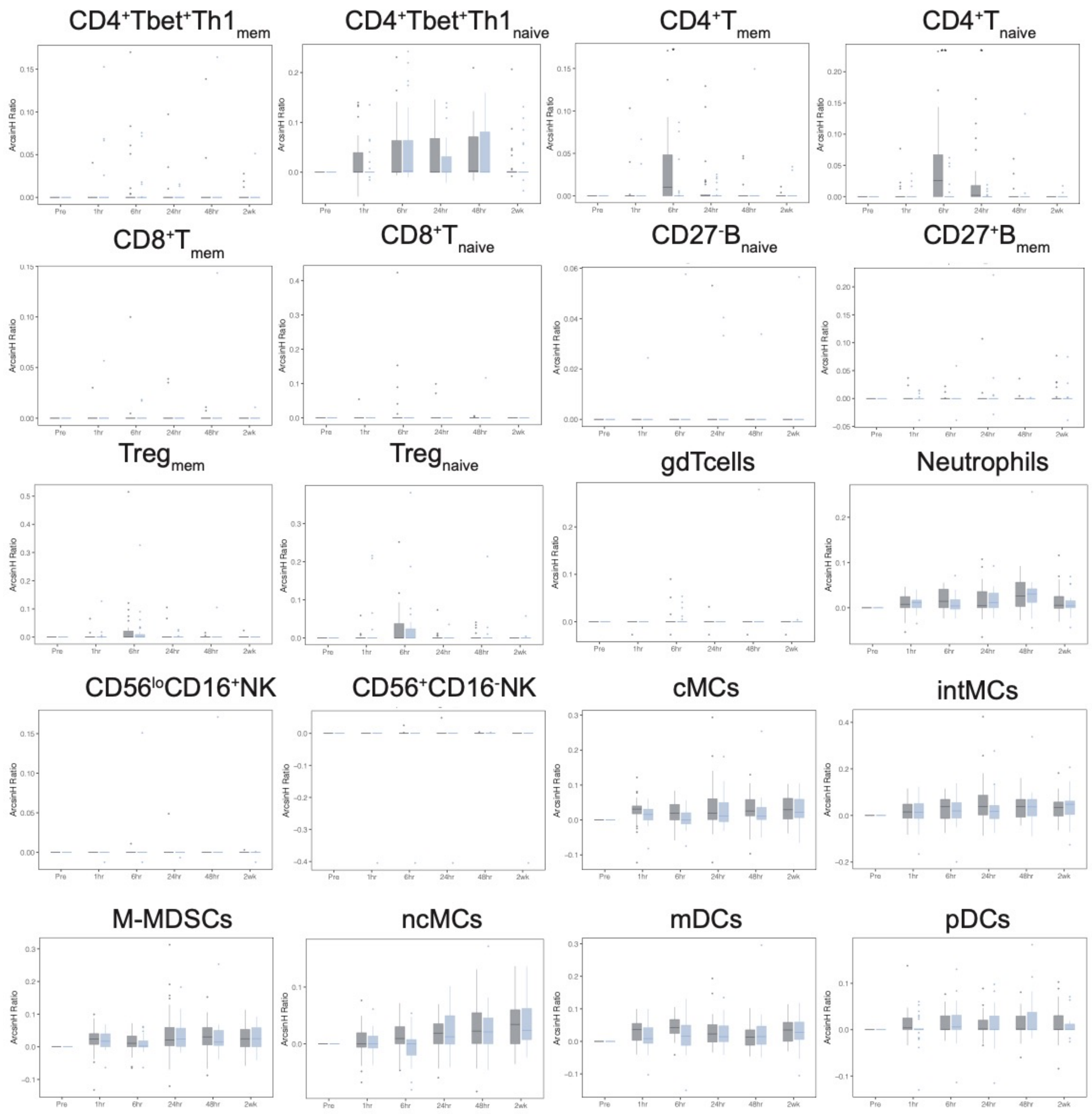

Supplementary Figure 12. Intracellular signaling response for phospho-Y701 STAT1 in manually gated cell subsets. Boxplots depict ArcSinh ratio over the endogenous signal response (y-axis) in 20 manually gated cell subsets before surgery (Pre) and 1h, 6h, 24h, 48h, and 2 weeks after surgery (x-axis) in control (n=30 patients; gray) and MP (n=28 patients; blue). All boxplots show median values, interquartile range, and whiskers of 1.5 times interquartile range. (Two-sided Wilcoxon rank-sum test. * = p<0.01, ** = p<0.001, *** = p<0.0001, p-values in **Supplementary Table 2**).

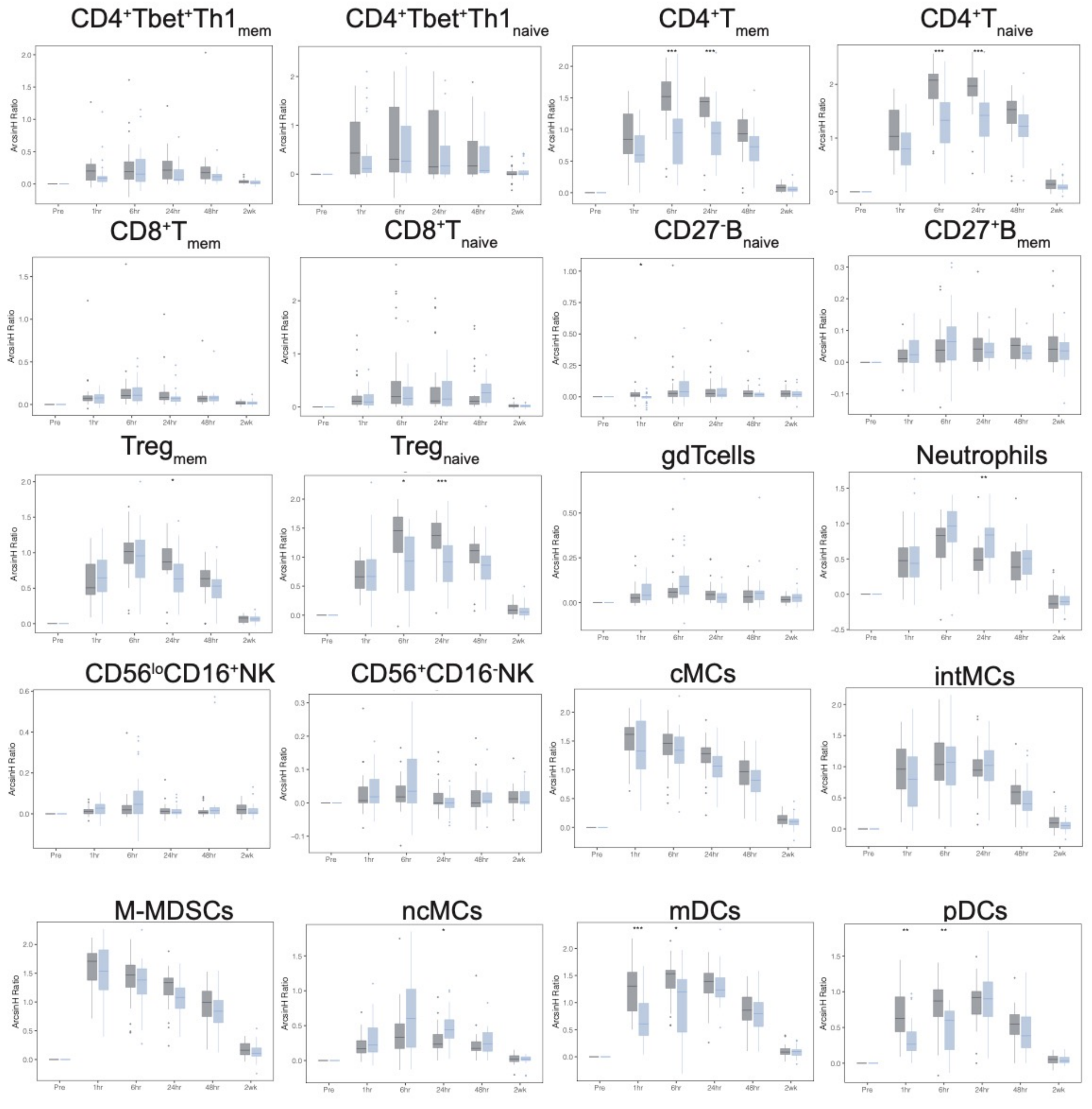

Supplementary Figure 13. Intracellular signaling response for phospho-Y705 STAT3 in manually gated cell subsets. Boxplots depict ArcSinh ratio over the endogenous signal response (y-axis) in 20 manually gated cell subsets before surgery (Pre) and 1h, 6h, 24h, 48h, and 2 weeks after surgery (x-axis) in control (n=30 patients; gray) and MP (n=28 patients; blue). All boxplots show median values, interquartile range, and whiskers of 1.5 times interquartile range. (Two-sided Wilcoxon rank-sum test. * = p<0.01, ** = p<0.001, *** = p<0.0001, p-values in **Supplementary Table 2**).

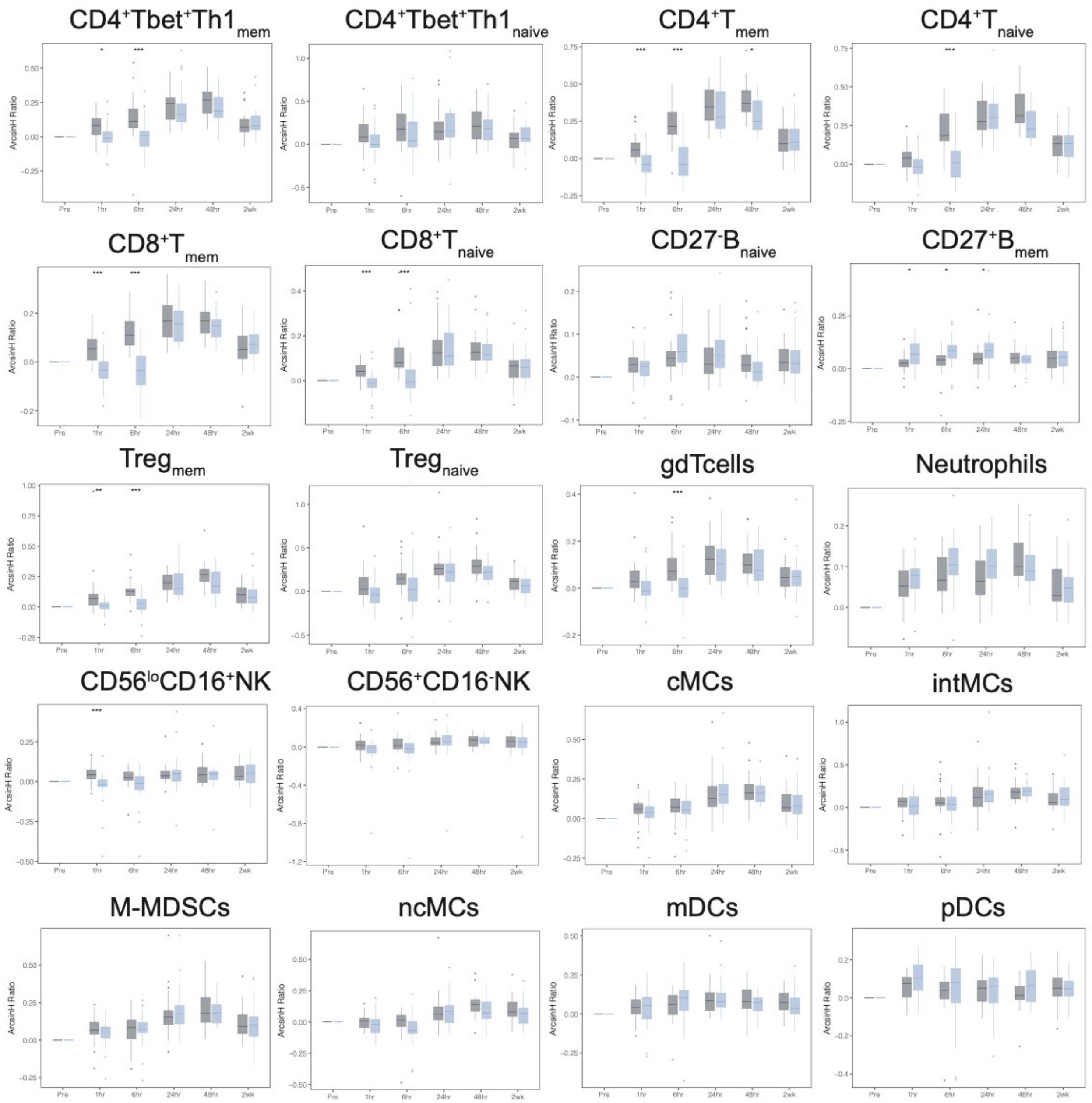

Supplementary Figure 14. Intracellular signaling response for phospho-Y694 STAT5 in manually gated cell subsets. Boxplots depict ArcSinh ratio over the endogenous signal response (y-axis) in 20 manually gated cell subsets before surgery (Pre) and 1h, 6h, 24h, 48h, and 2 weeks after surgery (x-axis) in control (n=30 patients; gray) and MP (n=28 patients; blue). All boxplots show median values, interquartile range, and whiskers of 1.5 times interquartile range. (Two-sided Wilcoxon rank-sum test. * = p<0.01, ** = p<0.001, *** = p<0.0001, p-values in **Supplementary Table 2**).

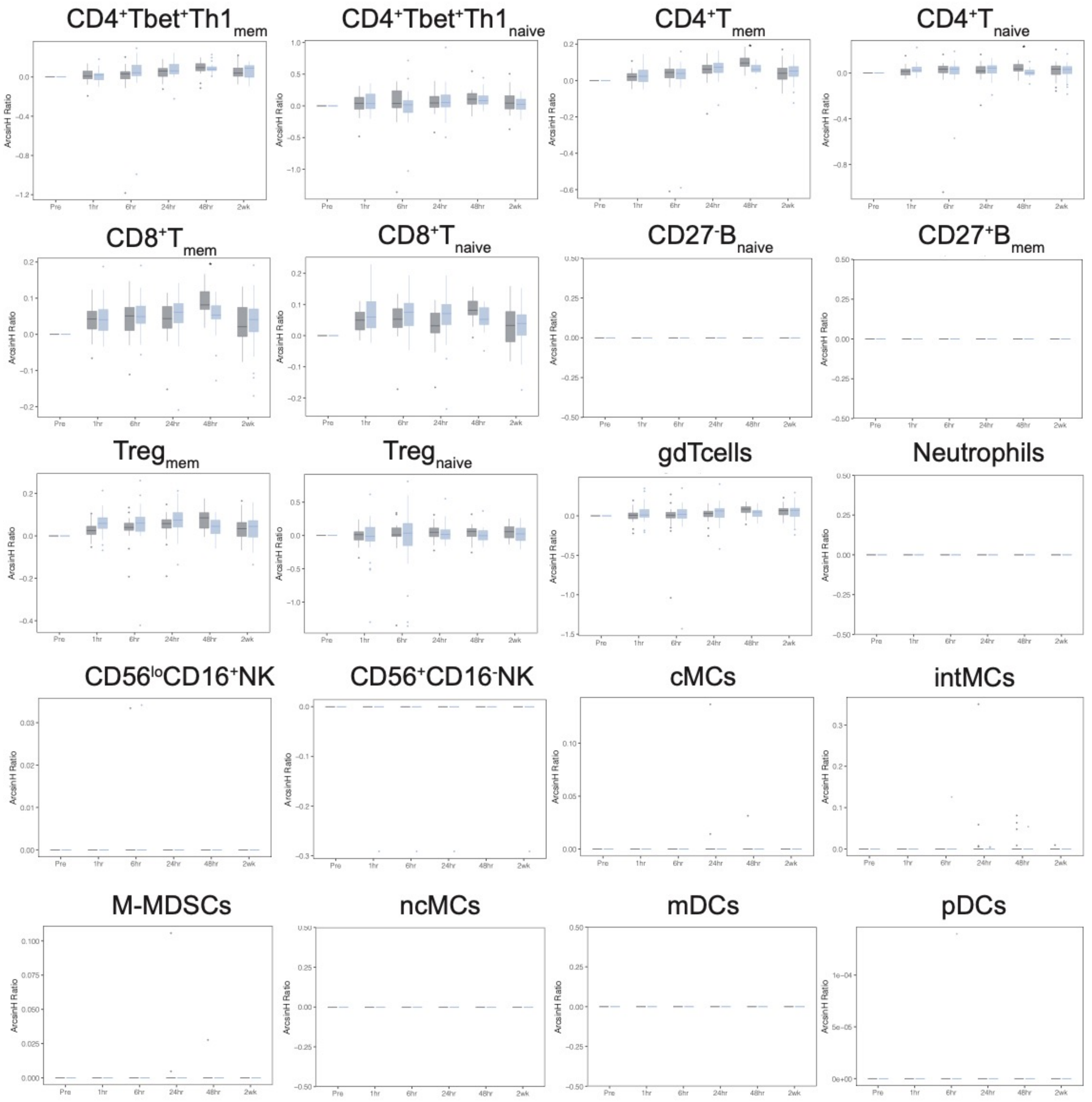

Supplementary Figure 15. Intracellular signaling response for phospho-Y641 STAT6 in manually gated cell subsets. Boxplots depict ArcSinh ratio over the endogenous signal response (y-axis) in 20 manually gated cell subsets before surgery (Pre) and 1h, 6h, 24h, 48h, and 2 weeks after surgery (x-axis) in control (n=30 patients; gray) and MP (n=28 patients; blue). All boxplots show median values, interquartile range, and whiskers of 1.5 times interquartile range. (Two-sided Wilcoxon rank-sum test. * = p<0.01, ** = p<0.001, *** = p<0.0001, p-values in **Supplementary Table 2**).

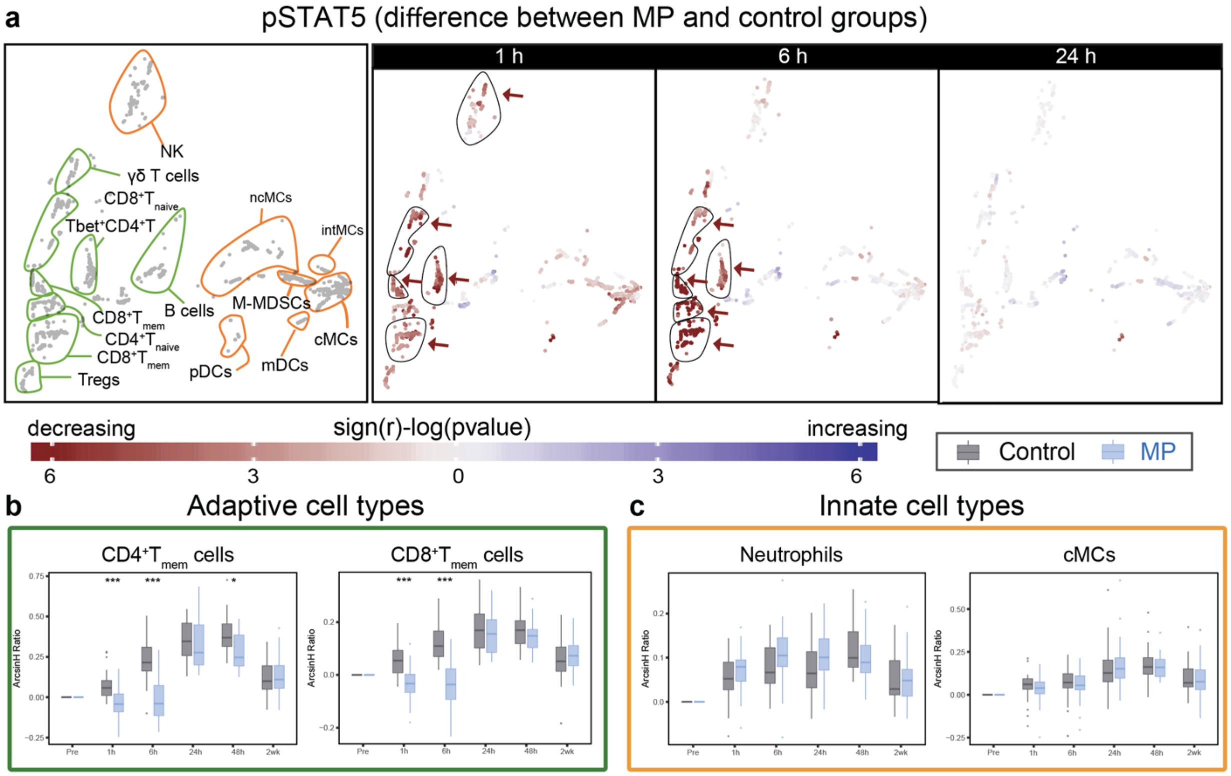

Supplementary Figure 16. Alteration of intracellular pSTAT5 responses by MP. A. Immune cell atlas depicting differences in the phospho-(p)STAT5 response (arcsinh ratio) between the MP (n=28 patients) and control (n=30 patients) group at 1, 6, and 24h after surgery relative to the preoperative time point. Blue/red cluster colors indicate increased/decreased signaling in the MP group, respectively (sign(r) -log(p-value), two-sided Wilcoxon rank-sum test). In adaptive cell clusters (contoured in green), MP treatment resulted in early attenuation of pSTAT5 responses in $CD4^+ T_{naive}$ cells at 6h, $CD4^+ T_{mem}$ cells at 1, 6, and 48h, and $CD8^+ T_{naive}$ and $CD8^+ T_{mem}$ cells at 1 and 6h. In innate cell clusters (contoured in orange), pSTAT5 signaling in $CD56^{lo}CD16^+$NK were attenuated at 1h. In contrast, MP resulted in no or minimal changes in the pSTAT5 signal in monocyte subsets (including cMCs, ncMCs, intMCs and M-MDSCs or mDCs. **B-C.** Box plots depict the pSTAT5 signal in manually gated immune cell subsets corroborating observations contained in the immune atlas. **B.** MP's attenuation of the pSTAT5 signal is most pronounced for $CD4^+ T_{mem}$ and $CD8^+ T_{mem}$ cell subsets. **C.** MP does not attenuate the pSTAT5 signal in neutrophils or cMCs. Boxplots show median values, interquartile range, and whiskers of 1.5 times interquartile range. (Two-sided Wilcoxon rank-sum test. * = p<0.01, ** = p<0.001, *** = p<0.0001, p-values in **Supplementary Table 2**).

Antibody	Manufacturer	Metal	Isotope	Clone	**Concentration**	Comment
Barcode 1	Trace Sciences	Pd	102		15µM	Barcode
Barcode 2	Trace Sciences	Pd	104		15µM	Barcode
Barcode 3	Trace Sciences	Pd	105		15µM	Barcode
Barcode 4	Trace Sciences	Pd	106		15µM	Barcode
Barcode 5	Trace Sciences	Pd	108		15µM	Barcode
Barcode 6	Trace Sciences	Pd	110		15µM	Barcode
CD235ab*	Biolegend	In	113	HIR2	1µg/mL	Phenotype
CD61*	BD	In	113	VI-PL2	0.5µg/mL	Phenotype
CD45	Biolegend	In	115	HI30	1µg/mL	Phenotype
CD66	BD	La	139	CD66a-B1.1	0.5µg/mL	Phenotype
CD7	BD	Pr	141	M-T701	0.5µg/mL	Phenotype
CD19	Biolegend	Nd	142	HIB19	0.5µg/mL	Phenotype
CD45RA	Biolegend	Nd	143	HI100	0.5µg/mL	Phenotype
CD11b	Biolegend	Nd	144	ICRF44	2µg/mL	Phenotype
CD4	Biolegend	Nd	145	RPA-T4	2µg/mL	Phenotype
CD8a	Biolegend	Nd	146	RPA-T8	1µg/mL	Phenotype
CD11c	Biolegend	Sm	147	Bu15	1µg/mL	Phenotype
CD123	Biolegend	Nd	148	6H6	1µg/mL	Phenotype
pCREB	Cell Signaling Technology	Sm	149	87G3	2µg/mL	Function
pSTAT5	Cell Signaling Technology	Nd	150	C11C5	4µg/mL	Function
pp38	BD	Eu	151	36/p38	2µg/mL	Function
TCRγδ	BD	Sm	152	B1	4µg/mL	Phenotype
pSTAT1	BD	Eu	153	14/P-STAT1	1µg/mL	Function
pSTAT3	Cell Signaling Technology	Sm	154	M9C6	2µg/mL	Function
pS6	Cell Signaling Technology	Gd	155	D57.2.2E	2µg/mL	Function
CD24	Biolegend	Gd	156	ML5	2µg/mL	Phenotype
CD161	Biolegend	Gd	157	HP-3G10	2µg/mL	Phenotype
CD33	Biolegend	Gd	158	WM53	2µg/mL	Phenotype
pMAPKAPK2	Cell Signaling Technology	Tb	159	27B7	1µg/mL	Function
Tbet	Thermo Fisher	Gd	160	4B10	8µg/mL	Phenotype
cPARP**	BD	Dy	161	F21-852	1µg/mL	Phenotype
FoxP3	Thermo Fisher	Dy	162	PCH101	8µg/mL	Phenotype
IκB	Cell Signaling Technology	Dy	164	L35A5	8µg/mL	Function
CD16	Biolegend	Ho	165	3G8	1µg/mL	Phenotype
pNFκB	BD	Er	166	K10-895.12.50	2µg/mL	Function
pERK1/2	Cell Signaling Technology	Er	167	D13.14.4E	4µg/mL	Function
pSTAT6	Biolegend	Er	168	A15137E	1µg/mL	Function
CD25	Biolegend	Tm	169	M-A251	2µg/mL	Phenotype
CD3	Biolegend	Er	170	UCHT1	1µg/mL	Phenotype
CD27	BD	Yb	171	M-T271	2µg/mL	Phenotype
CD15	Biolegend	Yb	172	W6D3	8µg/mL	Phenotype
CCR2	Biolegend	Yb	173	K036C2	2µg/mL	Phenotype
HLA-DR	Fluidigm	Yb	174	L243	2µg/mL	Phenotype
CD14	Fluidigm	Yb	175	M5E2	2µg/mL	Phenotype
CD56	BD	Yb	176	NCAM16.2	1µg/mL	Phenotype
DNA1†	Fluidigm	Ir	191		50µM	DNA
DNA2†	Fluidigm	Ir	192		50µM	DNA

Supplementary Table 1. Mass cytometry staining panel used in the study. *****Antibodies targeting CD235ab and CD61 were combined into the same channel in order to identify erythrocytes and platelets as a group for their removal (gating out) from subsequent analysis. ******D214-cleaved PARP (cPARP) was used to identify apoptotic and pre-apoptotic cells for their removal (gating out) from subsequent analysis. **†**An iridium-based DNA intercalator was used to help distinguish intact cells from cellular debris.

Cell type	Marker	Pre	1h	6h	24h	48h	2wk
CD27negBnaive	CREB	1.00E+00	3.63E-01	3.59E-01	7.35E-02	6.21E-01	2.74E-01
CD27posBmem	CREB	1.00E+00	7.86E-02	2.19E-01	1.17E-02	4.94E-01	3.37E-01
CD4TbetTh1mem	CREB	1.00E+00	6.43E-02	3.50E-01	4.15E-02	3.58E-01	5.29E-02
CD4TbetTh1naive	CREB	1.00E+00	5.23E-02	1.20E-01	7.09E-01	7.59E-01	7.24E-01
CD4Tmem	CREB	1.00E+00	5.34E-01	5.23E-01	1.81E-01	5.25E-01	1.21E-01
CD4Tnaive	CREB	1.00E+00	4.86E-01	7.20E-01	1.66E-01	3.70E-01	2.59E-01
CD56loCD16posNK	CREB	1.00E+00	9.24E-02	2.41E-03	3.05E-02	4.23E-01	1.73E-01
CD56posCD16negNK	CREB	1.00E+00	9.52E-01	2.50E-01	2.08E-01	5.56E-01	2.25E-01
CD8Tmem	CREB	1.00E+00	2.19E-01	3.43E-02	6.52E-01	5.88E-01	2.74E-01
CD8Tnaive	CREB	1.00E+00	8.12E-02	3.30E-02	1.97E-01	8.32E-01	1.48E-01
cMCs	CREB	1.00E+00	2.48E-02	5.13E-01	7.32E-01	7.24E-01	6.49E-01
gdTCells	CREB	1.00E+00	4.30E-01	1.28E-02	1.16E-01	8.14E-01	3.39E-02
Gr	CREB	1.00E+00	3.55E-01	6.74E-01	1.00E+00	4.94E-01	5.90E-01
intMCs	CREB	1.00E+00	1.26E-01	4.84E-01	4.27E-01	8.88E-01	4.00E-01
mDCs	CREB	1.00E+00	2.68E-01	7.67E-01	1.71E-01	4.37E-01	3.21E-01
MDSC	CREB	1.00E+00	1.08E-01	9.50E-01	5.65E-01	6.54E-01	7.36E-01
ncMCs	CREB	1.00E+00	9.95E-03	5.61E-03	9.88E-01	2.20E-01	1.21E-01
pDCs	CREB	1.00E+00	7.16E-01	1.52E-01	1.22E-02	3.00E-01	8.53E-01
Tregmem	CREB	1.00E+00	2.62E-01	2.02E-01	3.18E-02	1.44E-01	5.01E-01
Tregnaive	CREB	1.00E+00	9.88E-01	2.43E-01	6.63E-01	5.93E-02	4.34E-04
CD27negBnaive	ERK	1.00E+00	3.17E-01	6.63E-02	8.52E-01	7.32E-02	7.49E-01
CD27posBmem	ERK	1.00E+00	1.22E-01	1.27E-01	5.76E-02	4.03E-02	8.01E-01
CD4TbetTh1mem	ERK	1.00E+00	3.63E-01	7.20E-01	6.63E-01	2.89E-01	2.39E-01
CD4TbetTh1naive	ERK	1.00E+00	1.68E-01	9.13E-01	7.56E-01	6.89E-01	6.40E-02
CD4Tmem	ERK	1.00E+00	2.68E-01	2.41E-02	7.35E-02	2.09E-02	7.11E-01
CD4Tnaive	ERK	1.00E+00	7.97E-01	1.02E-01	2.02E-01	4.68E-03	5.33E-01
CD56loCD16posNK	ERK	1.00E+00	6.93E-01	5.75E-01	1.81E-01	7.95E-01	4.80E-01
CD56posCD16negNK	ERK	1.00E+00	4.67E-01	1.66E-01	1.31E-01	2.89E-01	1.62E-01
CD8Tmem	ERK	1.00E+00	9.40E-01	5.54E-01	1.02E-02	2.03E-01	2.74E-01
CD8Tnaive	ERK	1.00E+00	6.60E-01	2.02E-01	8.99E-02	2.03E-01	9.20E-01
cMCs	ERK	1.00E+00	6.71E-01	1.97E-01	2.63E-01	1.31E-01	9.87E-01
gdTCells	ERK	1.00E+00	9.15E-01	9.88E-01	2.08E-01	1.79E-01	4.29E-01
Gr	ERK	1.00E+00	9.82E-01	2.50E-02	1.57E-01	6.54E-01	9.06E-01
intMCs	ERK	1.00E+00	4.86E-01	8.40E-01	3.71E-02	6.95E-02	2.25E-01
mDCs	ERK	1.00E+00	4.37E-03	1.44E-01	9.26E-01	1.38E-01	8.93E-01
MDSC	ERK	1.00E+00	8.79E-01	4.81E-02	4.55E-01	1.87E-01	9.06E-01
ncMCs	ERK	1.00E+00	9.03E-01	8.28E-01	9.50E-01	5.88E-01	9.33E-01
pDCs	ERK	1.00E+00	2.82E-01	2.63E-01	9.50E-01	4.37E-01	5.78E-01

Tregmem	ERK	1.00E+00	1.09E-02	5.51E-04	4.00E-02	1.64E-01	5.78E-01
Tregnaive	ERK	1.00E+00	4.48E-01	1.44E-01	6.97E-01	1.44E-01	4.90E-01
CD27negBnaive	freq	6.08E-01	9.82E-03	5.03E-01	7.09E-01	2.20E-01	4.69E-01
CD27posBmem	freq	8.28E-01	7.16E-01	4.27E-01	8.76E-01	3.11E-01	9.33E-01
CD4TbetTh1mem	freq	8.52E-01	8.32E-01	8.09E-01	6.86E-01	9.25E-01	8.40E-01
CD4TbetTh1naive	freq	1.71E-01	5.05E-01	9.69E-01	8.40E-01	5.25E-01	2.59E-01
CD4Tmem	freq	6.41E-02	8.32E-07	9.44E-08	8.70E-02	5.04E-02	1.02E-01
CD4Tnaive	freq	9.88E-01	5.42E-02	8.21E-04	6.08E-01	1.57E-01	8.80E-01
CD56loCD16posNK	freq	4.10E-01	5.03E-03	2.50E-02	6.63E-02	8.11E-02	1.17E-01
CD56posCD16negNK	freq	9.50E-01	4.39E-01	9.26E-01	3.84E-01	3.83E-01	4.29E-01
CD8Tmem	freq	4.65E-01	9.88E-01	6.30E-01	8.28E-01	8.14E-01	4.80E-01
CD8Tnaive	freq	4.31E-02	6.06E-03	8.14E-02	6.87E-02	3.11E-01	5.29E-02
cMCs	freq	2.97E-01	2.54E-07	3.75E-01	6.74E-01	9.67E-04	6.74E-01
gdTCells	freq	9.88E-01	1.59E-01	5.23E-01	9.01E-01	6.71E-01	6.02E-01
Gr	freq	4.46E-01	2.02E-08	7.93E-10	1.52E-01	2.29E-01	2.07E-01
intMCs	freq	6.30E-01	9.88E-01	5.65E-01	9.13E-01	1.26E-01	7.49E-01
mDCs	freq	5.03E-01	2.58E-04	2.24E-06	2.55E-04	2.68E-01	1.73E-01
MDSC	freq	3.04E-01	1.24E-02	3.80E-03	9.38E-01	1.50E-01	9.73E-01
ncMCs	freq	6.52E-01	2.31E-01	5.34E-01	1.59E-02	7.32E-02	9.33E-01
pDCs	freq	4.65E-01	2.12E-02	3.40E-06	1.47E-04	5.03E-05	5.78E-01
Tregmem	freq	2.94E-02	1.53E-02	5.37E-02	7.87E-02	3.00E-01	3.22E-03
Tregnaive	freq	7.91E-01	3.13E-02	1.42E-02	3.84E-01	9.62E-01	1.73E-01
CD27negBnaive	IkB	1.00E+00	7.50E-01	5.10E-03	4.15E-02	6.37E-01	2.39E-01
CD27posBmem	IkB	1.00E+00	5.44E-01	5.23E-01	8.14E-02	6.71E-01	3.21E-01
CD4TbetTh1mem	IkB	1.00E+00	2.55E-01	2.29E-03	1.28E-03	3.58E-01	3.46E-01
CD4TbetTh1naive	IkB	1.00E+00	4.95E-01	1.44E-01	4.31E-02	7.77E-01	3.46E-01
CD4Tmem	IkB	1.00E+00	7.05E-01	1.61E-01	1.03E-03	9.42E-02	4.59E-01
CD4Tnaive	IkB	1.00E+00	7.85E-01	2.04E-02	1.52E-02	1.64E-01	4.19E-01
CD56loCD16posNK	IkB	1.00E+00	4.36E-04	3.06E-07	1.50E-03	3.70E-01	4.69E-01
CD56posCD16negNK	IkB	1.00E+00	2.48E-02	1.35E-03	3.11E-03	6.04E-01	7.11E-01
CD8Tmem	IkB	1.00E+00	2.89E-01	2.41E-02	4.27E-05	2.84E-02	4.69E-01
CD8Tnaive	IkB	1.00E+00	5.24E-01	3.05E-02	5.57E-05	3.47E-03	5.56E-01
cMCs	IkB	1.00E+00	2.58E-04	1.86E-07	2.82E-07	3.70E-01	7.36E-01
gdTCells	IkB	1.00E+00	2.08E-01	3.44E-03	6.36E-05	3.70E-01	5.33E-01
Gr	IkB	1.00E+00	3.24E-06	2.20E-07	1.66E-04	4.51E-01	9.06E-01
intMCs	IkB	1.00E+00	2.79E-02	2.13E-04	3.45E-04	4.51E-01	1.14E-01
mDCs	IkB	1.00E+00	2.68E-01	2.04E-02	4.77E-06	4.51E-01	7.24E-01
MDSC	IkB	1.00E+00	3.26E-04	7.94E-08	1.92E-06	1.72E-01	1.00E+00
ncMCs	IkB	1.00E+00	7.12E-07	2.60E-07	4.06E-09	5.88E-01	4.19E-01

pDCs	IkB	1.00E+00	6.60E-01	3.12E-01	3.45E-04	6.37E-01	9.60E-01
Tregmem	IkB	1.00E+00	7.27E-01	1.20E-01	6.17E-03	4.09E-01	4.09E-01
Tregnaive	IkB	1.00E+00	8.08E-01	4.10E-01	3.30E-02	3.11E-01	6.49E-01
CD27negBnaive	MAPKAPK2	1.00E+00	5.53E-03	4.31E-02	4.84E-01	7.41E-01	8.53E-01
CD27posBmem	MAPKAPK2	1.00E+00	3.38E-02	1.66E-01	4.10E-01	8.88E-01	8.66E-01
CD4TbetTh1mem	MAPKAPK2	1.00E+00	5.23E-02	2.31E-01	1.27E-01	7.06E-01	9.60E-01
CD4TbetTh1naive	MAPKAPK2	1.00E+00	1.08E-01	4.27E-01	6.19E-02	2.20E-01	6.40E-02
CD4Tmem	MAPKAPK2	1.00E+00	2.69E-02	4.64E-02	1.81E-01	8.32E-01	8.93E-01
CD4Tnaive	MAPKAPK2	1.00E+00	5.61E-02	9.29E-02	4.18E-01	6.21E-01	9.87E-01
CD56loCD16posNK	MAPKAPK2	1.00E+00	1.08E-01	1.52E-02	4.94E-01	2.58E-01	7.36E-01
CD56posCD16negNK	MAPKAPK2	1.00E+00	8.95E-02	3.57E-02	4.37E-01	8.88E-01	7.24E-01
CD8Tmem	MAPKAPK2	1.00E+00	9.52E-03	2.04E-02	2.76E-01	6.21E-01	6.02E-01
CD8Tnaive	MAPKAPK2	1.00E+00	2.69E-02	8.55E-03	2.50E-01	4.09E-01	4.19E-01
cMCs	MAPKAPK2	1.00E+00	7.12E-02	4.10E-01	2.61E-02	2.48E-01	8.66E-01
gdTCells	MAPKAPK2	1.00E+00	2.75E-01	1.66E-01	3.04E-01	2.78E-01	6.25E-01
Gr	MAPKAPK2	1.00E+00	7.39E-01	3.57E-02	2.13E-01	3.20E-02	8.93E-01
intMCs	MAPKAPK2	1.00E+00	4.87E-02	4.64E-02	8.76E-01	2.68E-01	6.37E-01
mDCs	MAPKAPK2	1.00E+00	8.68E-01	9.75E-01	2.71E-02	9.44E-01	9.46E-01
MDSC	MAPKAPK2	1.00E+00	1.68E-01	4.27E-01	2.82E-02	4.80E-01	5.22E-01
ncMCs	MAPKAPK2	1.00E+00	2.58E-02	7.76E-04	9.88E-01	1.09E-02	6.25E-01
pDCs	MAPKAPK2	1.00E+00	8.68E-01	4.84E-01	5.23E-01	7.41E-01	7.75E-01
Tregmem	MAPKAPK2	1.00E+00	1.05E-01	6.41E-01	8.70E-02	9.44E-01	9.06E-01
Tregnaive	MAPKAPK2	1.00E+00	1.08E-01	8.03E-01	8.52E-01	8.88E-01	5.44E-01
CD27negBnaive	NFkB	1.00E+00	1.09E-02	1.39E-02	4.55E-01	2.89E-01	5.67E-01
CD27posBmem	NFkB	1.00E+00	6.35E-03	3.30E-02	3.92E-01	7.77E-01	7.62E-01
CD4TbetTh1mem	NFkB	1.00E+00	8.72E-03	1.73E-02	6.74E-01	8.50E-01	4.00E-01
CD4TbetTh1naive	NFkB	1.00E+00	2.29E-02	2.50E-02	6.30E-01	6.89E-01	6.86E-01
CD4Tmem	NFkB	1.00E+00	3.43E-03	2.50E-02	9.75E-01	4.80E-01	9.60E-01
CD4Tnaive	NFkB	1.00E+00	6.06E-03	1.46E-02	9.63E-01	5.56E-01	8.80E-01
CD56loCD16posNK	NFkB	1.00E+00	3.78E-03	3.85E-02	6.19E-01	3.22E-01	5.90E-01
CD56posCD16negNK	NFkB	1.00E+00	4.80E-03	3.99E-03	5.54E-01	9.62E-01	8.27E-01
CD8Tmem	NFkB	1.00E+00	2.31E-03	7.10E-03	8.52E-01	9.25E-01	6.99E-01
CD8Tnaive	NFkB	1.00E+00	2.96E-03	2.17E-03	8.40E-01	6.71E-01	5.33E-01
cMCs	NFkB	1.00E+00	1.18E-01	8.03E-01	7.44E-01	1.57E-01	8.80E-01
gdTCells	NFkB	1.00E+00	1.88E-02	1.96E-02	6.86E-01	8.32E-01	5.33E-01
Gr	NFkB	1.00E+00	5.14E-01	4.15E-02	3.05E-02	8.69E-01	8.80E-01
intMCs	NFkB	1.00E+00	1.02E-01	7.09E-01	9.63E-01	3.83E-01	2.89E-01
mDCs	NFkB	1.00E+00	9.52E-01	4.15E-02	6.97E-01	2.68E-01	5.56E-01
MDSC	NFkB	1.00E+00	2.49E-01	4.37E-01	8.76E-01	1.72E-01	7.62E-01

ncMCs	NFkB	1.00E+00	7.61E-02	3.99E-03	7.32E-01	8.11E-02	3.72E-01
pDCs	NFkB	1.00E+00	7.36E-02	2.02E-01	7.87E-02	3.22E-01	8.01E-01
Tregmem	NFkB	1.00E+00	7.86E-02	1.91E-01	2.83E-01	4.94E-01	8.93E-01
Tregnaive	NFkB	1.00E+00	5.96E-01	9.38E-01	3.04E-01	4.09E-01	8.27E-01
CD27negBnaive	p38	1.00E+00	1.00E+00	9.63E-02	4.89E-01	8.43E-01	4.42E-01
CD27posBmem	p38	1.00E+00	1.77E-01	1.22E-01	4.41E-01	1.88E-01	9.07E-01
CD4TbetTh1mem	p38	1.00E+00	1.61E-01	9.29E-01	1.26E-01	1.92E-01	9.26E-01
CD4TbetTh1naive	p38	1.00E+00	2.17E-01	4.92E-01	9.85E-01	4.00E-01	3.90E-01
CD4Tmem	p38	1.00E+00	5.50E-01	6.78E-01	4.78E-01	4.41E-01	7.14E-01
CD4Tnaive	p38	1.00E+00	1.61E-01	9.86E-01	1.96E-01	1.92E-01	7.36E-01
CD56loCD16posNK	p38	1.00E+00	5.64E-01	1.61E-01	9.86E-01	9.22E-01	6.85E-01
CD56posCD16negNK	p38	1.00E+00	1.00E+00	5.43E-01	3.30E-01	1.00E+00	1.00E+00
CD8Tmem	p38	1.00E+00	1.61E-01	5.70E-01	3.57E-01	3.61E-01	7.10E-01
CD8Tnaive	p38	1.00E+00	1.61E-01	1.61E-01	5.17E-01	3.61E-01	7.10E-01
cMCs	p38	1.00E+00	6.57E-01	1.04E-01	6.12E-01	7.23E-01	8.76E-01
gdTCells	p38	1.00E+00	3.09E-01	1.61E-01	3.57E-01	1.00E+00	1.00E+00
Gr	p38	1.00E+00	5.64E-01	1.00E+00	7.11E-01	4.29E-01	6.52E-01
intMCs	p38	1.00E+00	3.31E-01	4.65E-01	9.57E-01	8.69E-01	3.31E-01
mDCs	p38	1.00E+00	6.96E-01	4.99E-02	3.69E-01	2.33E-01	8.59E-01
MDSC	p38	1.00E+00	9.38E-01	5.49E-02	7.08E-01	6.79E-01	9.44E-01
ncMCs	p38	1.00E+00	5.82E-01	4.18E-01	4.53E-01	6.17E-01	5.76E-01
pDCs	p38	1.00E+00	6.84E-01	4.79E-03	1.16E-02	4.23E-01	7.57E-01
Tregmem	p38	1.00E+00	5.64E-01	6.32E-01	3.85E-01	1.92E-01	9.26E-01
Tregnaive	p38	1.00E+00	3.86E-01	3.77E-01	6.49E-01	5.21E-01	9.75E-01
CD27negBnaive	pSTAT6	1.00E+00	1.00E+00	1.00E+00	1.00E+00	1.00E+00	1.00E+00
CD27posBmem	pSTAT6	1.00E+00	1.00E+00	1.00E+00	1.00E+00	1.00E+00	1.00E+00
CD4TbetTh1mem	pSTAT6	1.00E+00	9.28E-01	6.63E-02	1.27E-01	4.23E-01	2.39E-01
CD4TbetTh1naive	pSTAT6	1.00E+00	9.15E-01	2.31E-01	9.50E-01	6.89E-01	4.29E-01
CD4Tmem	pSTAT6	1.00E+00	8.56E-01	6.86E-01	5.44E-01	3.74E-03	6.37E-01
CD4Tnaive	pSTAT6	1.00E+00	2.19E-01	7.56E-01	3.67E-01	5.03E-03	7.36E-01
CD56loCD16posNK	pSTAT6	1.00E+00	1.00E+00	9.41E-01	1.00E+00	1.00E+00	1.00E+00
CD56posCD16negNK	pSTAT6	1.00E+00	3.09E-01	3.01E-01	3.01E-01	1.00E+00	3.09E-01
CD8Tmem	pSTAT6	1.00E+00	7.62E-01	9.63E-01	4.74E-01	8.29E-03	5.90E-01
CD8Tnaive	pSTAT6	1.00E+00	2.31E-01	2.19E-01	5.37E-02	3.59E-02	7.62E-01
cMCs	pSTAT6	1.00E+00	1.00E+00	1.00E+00	1.68E-01	3.61E-01	1.00E+00
gdTCells	pSTAT6	1.00E+00	4.13E-01	5.97E-01	2.97E-01	2.51E-02	9.33E-01
Gr	pSTAT6	1.00E+00	1.00E+00	1.00E+00	1.00E+00	1.00E+00	1.00E+00
intMCs	pSTAT6	1.00E+00	1.00E+00	3.01E-01	3.77E-01	2.31E-01	3.26E-01
mDCs	pSTAT6	1.00E+00	1.00E+00	1.00E+00	1.00E+00	1.00E+00	1.00E+00

MDSC	pSTAT6	1.00E+00	1.00E+00	1.00E+00	1.68E-01	3.61E-01	1.00E+00
ncMCs	pSTAT6	1.00E+00	1.00E+00	1.00E+00	1.00E+00	1.00E+00	1.00E+00
pDCs	pSTAT6	1.00E+00	1.00E+00	3.01E-01	1.00E+00	1.00E+00	1.00E+00
Tregmem	pSTAT6	1.00E+00	1.80E-02	1.86E-01	8.99E-02	1.84E-02	5.90E-01
Tregnaive	pSTAT6	1.00E+00	8.44E-01	9.88E-01	4.10E-01	1.14E-01	3.91E-01
CD27negBnaive	S6	1.00E+00	1.88E-03	1.09E-01	5.76E-02	2.12E-01	6.90E-02
CD27posBmem	S6	1.00E+00	7.97E-01	6.41E-01	8.40E-01	5.56E-01	2.13E-01
CD4TbetTh1mem	S6	1.00E+00	4.37E-02	1.39E-01	1.27E-01	1.09E-01	9.60E-01
CD4TbetTh1naive	S6	1.00E+00	5.44E-01	7.91E-01	1.61E-01	1.20E-01	7.49E-01
CD4Tmem	S6	1.00E+00	6.71E-01	8.89E-01	3.27E-01	1.44E-01	3.91E-01
CD4Tnaive	S6	1.00E+00	3.17E-01	7.20E-01	1.78E-01	4.15E-02	2.04E-01
CD56loCD16posNK	S6	1.00E+00	3.01E-02	7.44E-01	6.30E-01	8.88E-01	7.88E-01
CD56posCD16negNK	S6	1.00E+00	7.44E-01	1.07E-01	7.62E-01	4.23E-01	3.16E-01
CD8Tmem	S6	1.00E+00	1.31E-01	1.02E-02	1.31E-01	6.29E-01	7.49E-01
CD8Tnaive	S6	1.00E+00	7.94E-03	1.52E-02	3.18E-02	9.16E-01	1.06E-01
cMCs	S6	1.00E+00	7.85E-01	9.50E-01	1.39E-01	7.95E-01	5.56E-01
gdTCells	S6	1.00E+00	6.82E-01	6.08E-01	6.19E-01	7.24E-01	7.62E-01
Gr	S6	1.00E+00	2.08E-01	2.90E-01	2.97E-01	2.39E-01	5.11E-01
intMCs	S6	1.00E+00	4.39E-01	7.91E-01	5.97E-01	7.06E-01	1.95E-01
mDCs	S6	1.00E+00	5.96E-01	8.03E-01	7.79E-01	1.95E-01	4.51E-02
MDSC	S6	1.00E+00	9.28E-01	3.12E-01	6.41E-02	6.71E-01	9.46E-01
ncMCs	S6	1.00E+00	9.88E-01	2.31E-02	3.35E-01	1.79E-01	9.06E-01
pDCs	S6	1.00E+00	1.92E-01	4.46E-01	1.44E-01	4.80E-01	7.88E-01
Tregmem	S6	1.00E+00	9.54E-02	4.64E-02	1.61E-01	7.59E-01	1.00E+00
Tregnaive	S6	1.00E+00	6.71E-01	3.27E-01	1.00E+00	5.09E-01	9.33E-01
CD27negBnaive	STAT1	1.00E+00	3.09E-01	3.01E-01	5.43E-01	2.73E-01	3.09E-01
CD27posBmem	STAT1	1.00E+00	7.26E-01	2.13E-01	6.40E-01	6.27E-01	4.99E-01
CD4TbetTh1mem	STAT1	1.00E+00	1.44E-01	5.90E-01	6.78E-01	7.06E-01	3.50E-01
CD4TbetTh1naive	STAT1	1.00E+00	1.07E-01	7.47E-01	5.50E-01	8.50E-01	9.34E-01
CD4Tmem	STAT1	1.00E+00	6.72E-01	3.87E-03	5.62E-01	4.36E-01	9.11E-01
CD4Tnaive	STAT1	1.00E+00	7.46E-01	3.24E-04	3.63E-03	7.97E-01	1.46E-01
CD56loCD16posNK	STAT1	1.00E+00	3.09E-01	5.98E-01	1.61E-01	2.73E-01	5.64E-01
CD56posCD16negNK	STAT1	1.00E+00	3.09E-01	8.86E-02	1.61E-01	9.22E-01	3.09E-01
CD8Tmem	STAT1	1.00E+00	9.61E-01	9.44E-01	1.68E-01	7.06E-01	3.09E-01
CD8Tnaive	STAT1	1.00E+00	3.26E-01	2.53E-02	1.68E-01	7.06E-01	1.00E+00
cMCs	STAT1	1.00E+00	4.85E-02	3.05E-02	3.12E-01	2.12E-01	9.60E-01
gdTCells	STAT1	1.00E+00	3.26E-01	3.11E-01	1.00E+00	1.60E-01	1.61E-01
Gr	STAT1	1.00E+00	8.43E-01	7.93E-02	8.27E-01	9.72E-01	6.07E-01
intMCs	STAT1	1.00E+00	7.50E-01	3.92E-01	2.63E-01	7.24E-01	5.67E-01

mDCs	STAT1	1.00E+00	1.69E-01	4.47E-02	5.54E-01	9.16E-01	9.33E-01
MDSC	STAT1	1.00E+00	3.83E-01	2.96E-01	7.03E-01	3.52E-01	6.80E-01
ncMCs	STAT1	1.00E+00	6.85E-01	4.02E-02	8.89E-01	6.04E-01	9.33E-01
pDCs	STAT1	1.00E+00	6.25E-02	7.26E-01	4.76E-01	9.71E-01	7.80E-01
Tregmem	STAT1	1.00E+00	3.86E-01	4.69E-01	6.55E-01	7.06E-01	3.26E-01
Tregnaive	STAT1	1.00E+00	5.62E-01	9.85E-01	2.01E-01	8.82E-01	1.46E-01
CD27negBnaive	STAT3	1.00E+00	4.11E-03	4.44E-01	8.26E-01	4.03E-01	5.99E-01
CD27posBmem	STAT3	1.00E+00	5.95E-01	1.57E-01	7.32E-01	1.34E-01	6.98E-01
CD4TbetTh1mem	STAT3	1.00E+00	3.56E-02	4.64E-01	4.63E-02	1.47E-01	2.12E-01
CD4TbetTh1naive	STAT3	1.00E+00	4.25E-01	8.76E-01	3.74E-01	7.50E-01	5.55E-01
CD4Tmem	STAT3	1.00E+00	1.04E-02	6.36E-05	2.31E-05	1.96E-02	3.12E-01
CD4Tnaive	STAT3	1.00E+00	3.13E-02	4.27E-05	3.26E-05	4.51E-02	1.67E-01
CD56loCD16posNK	STAT3	1.00E+00	1.53E-01	1.06E-01	5.63E-01	3.87E-01	4.16E-01
CD56posCD16negNK	STAT3	1.00E+00	9.51E-01	6.29E-01	1.46E-01	7.58E-01	7.10E-01
CD8Tmem	STAT3	1.00E+00	8.32E-01	7.15E-01	1.88E-01	7.77E-01	9.33E-01
CD8Tnaive	STAT3	1.00E+00	3.95E-01	5.44E-01	8.95E-01	2.19E-01	8.00E-01
cMCs	STAT3	1.00E+00	3.47E-01	5.03E-01	2.50E-02	1.95E-01	3.12E-01
gdTCells	STAT3	1.00E+00	1.04E-01	9.13E-02	1.94E-01	2.99E-01	2.19E-01
Gr	STAT3	1.00E+00	5.75E-01	2.94E-02	8.69E-04	2.68E-01	5.96E-01
intMCs	STAT3	1.00E+00	1.22E-01	8.52E-01	9.38E-01	3.34E-01	1.14E-01
mDCs	STAT3	1.00E+00	4.24E-05	1.96E-03	4.01E-01	5.56E-01	8.14E-01
MDSC	STAT3	1.00E+00	5.85E-01	3.59E-01	2.71E-02	2.58E-01	2.74E-01
ncMCs	STAT3	1.00E+00	3.71E-01	3.71E-02	2.41E-03	3.58E-01	8.53E-01
pDCs	STAT3	1.00E+00	2.03E-04	3.06E-04	5.86E-01	1.50E-01	7.36E-01
Tregmem	STAT3	1.00E+00	6.28E-01	4.74E-01	1.86E-03	8.11E-02	7.88E-01
Tregnaive	STAT3	1.00E+00	8.44E-01	1.58E-03	4.88E-05	3.81E-02	1.95E-01
CD27negBnaive	STAT5	1.00E+00	5.14E-01	1.23E-01	2.13E-01	1.31E-01	6.99E-01
CD27posBmem	STAT5	1.00E+00	7.28E-03	4.63E-03	4.63E-03	4.23E-01	5.67E-01
CD4TbetTh1mem	STAT5	1.00E+00	1.45E-03	4.56E-05	4.10E-01	1.14E-01	4.00E-01
CD4TbetTh1naive	STAT5	1.00E+00	3.25E-02	4.64E-02	9.01E-01	7.41E-01	5.22E-01
CD4Tmem	STAT5	1.00E+00	3.72E-05	2.02E-07	3.50E-01	7.21E-03	6.49E-01
CD4Tnaive	STAT5	1.00E+00	2.39E-02	2.60E-07	9.01E-01	1.02E-02	9.60E-01
CD56loCD16posNK	STAT5	1.00E+00	1.66E-05	1.80E-02	7.79E-01	9.25E-01	9.46E-01
CD56posCD16negNK	STAT5	1.00E+00	4.07E-02	1.34E-02	8.40E-01	9.06E-01	8.27E-01
CD8Tmem	STAT5	1.00E+00	9.54E-06	7.29E-08	5.13E-01	3.34E-01	5.11E-01
CD8Tnaive	STAT5	1.00E+00	3.26E-05	2.31E-05	7.20E-01	7.77E-01	8.80E-01
cMCs	STAT5	1.00E+00	4.04E-01	6.30E-01	4.37E-01	7.06E-01	8.27E-01
gdTCells	STAT5	1.00E+00	1.40E-02	8.26E-05	2.97E-01	3.00E-01	9.33E-01
Gr	STAT5	1.00E+00	1.97E-01	5.76E-02	2.50E-02	3.83E-01	7.88E-01

intMCs	STAT5	1.00E+00	2.43E-01	3.67E-01	7.20E-01	6.89E-01	4.59E-01
mDCs	STAT5	1.00E+00	7.62E-01	1.76E-01	6.52E-01	3.11E-01	3.04E-01
MDSC	STAT5	1.00E+00	3.47E-01	9.38E-01	4.94E-01	7.24E-01	6.49E-01
ncMCs	STAT5	1.00E+00	1.72E-01	1.46E-02	7.67E-01	2.58E-01	3.91E-01
pDCs	STAT5	1.00E+00	1.15E-01	2.63E-01	9.01E-01	2.78E-01	8.80E-01
Tregmem	STAT5	1.00E+00	5.46E-04	4.11E-06	4.27E-01	5.33E-02	5.90E-01
Tregnaive	STAT5	1.00E+00	3.78E-02	2.04E-02	4.37E-01	7.32E-02	1.34E-01

Supplementary Table 2. Two-sided Wilcoxon rank sum test p-values. Wilcoxon rank sum tests were computed using IBM SPSS Statistics, version 26.0.0.1.
