# Supplementary material for: Preferential inhibition of adaptive immune system dynamics by glucocorticoids in patients after acute surgical trauma
Source: Nat Commun. 2020 Jul 27;11:3737. doi: 10.1038/s41467-020-17565-y (PMC7385146; doi:10.1038/s41467-020-17565-y)
Supplement: Supplementary file 3 — Reporting Summary [file 41467_2020_17565_MOESM3_ESM.pdf]

nature research

Corresponding author(s): Brice Gaudilliere

Last updated by author(s): 05-15-2020

Reporting Summary

Nature Research wishes to improve the reproducibility of the work that we publish. This form provides structure for consistency and transparency in reporting. For further information on Nature Research policies, see Authors & Referees and the Editorial Policy Checklist.

Statistics

For all statistical analyses, confirm that the following items are present in the figure legend, table legend, main text, or Methods section.

n/a	Confirmed	
☐	☒	The exact sample size (n) for each experimental group/condition, given as a discrete number and unit of measurement
☐	☒	A statement on whether measurements were taken from distinct samples or whether the same sample was measured repeatedly
☐	☒	The statistical test(s) used AND whether they are one- or two-sided<br>*Only common tests should be described solely by name; describe more complex techniques in the Methods section.*
☐	☒	A description of all covariates tested
☐	☒	A description of any assumptions or corrections, such as tests of normality and adjustment for multiple comparisons
☐	☒	A full description of the statistical parameters including central tendency (e.g. means) or other basic estimates (e.g. regression coefficient) AND variation (e.g. standard deviation) or associated estimates of uncertainty (e.g. confidence intervals)
☐	☒	For null hypothesis testing, the test statistic (e.g. F, t, r) with confidence intervals, effect sizes, degrees of freedom and P value noted<br>*Give P values as exact values whenever suitable.*
☒	☐	For Bayesian analysis, information on the choice of priors and Markov chain Monte Carlo settings
☐	☒	For hierarchical and complex designs, identification of the appropriate level for tests and full reporting of outcomes
☒	☐	Estimates of effect sizes (e.g. Cohen's d, Pearson's r), indicating how they were calculated

Our web collection on statistics for biologists contains articles on many of the points above.

Software and code

Policy information about availability of computer code

Data collection	Mass cytometry FCS files were normalized and debarcoded using Matlab 9.7 based software available at https://github.com/ParkerICI premessa<br>The resulting FCS files were uploaded to the Cell Engine (https://cellengine.com, Primity Bio, Fremont, CA) flow cytometry analysis platform.<br>Other softwares (CITRUS, FLOWSOM, Phenograph) mentioned in the text with references to initial publications were not used in the analysis.
Data analysis	Source code for data analysis is available upon request at http://stanford.edu/~stanleyn/Steroid_Immune/

For manuscripts utilizing custom algorithms or software that are central to the research but not yet described in published literature, software must be made available to editors/reviewers. We strongly encourage code deposition in a community repository (e.g. GitHub). See the Nature Research guidelines for submitting code & software for further information.

Data

Policy information about availability of data

All manuscripts must include a data availability statement. This statement should provide the following information, where applicable:

- Accession codes, unique identifiers, or web links for publicly available datasets
- A list of figures that have associated raw data
- A description of any restrictions on data availability

Raw data necessary to reproduce all figures were uploaded and made publicly available at https://flowrepository.org/id/FR-FCM-Z2AT.
Data necessary to reproduce bar graphs in Fig 1 and 3-5 and Fig. S4,S5 is attached to this submission: Ganio et al_Source_Data_R2.xlsx

Field-specific reporting

Please select the one below that is the best fit for your research. If you are not sure, read the appropriate sections before making your selection.

☒ Life sciences ☐ Behavioural & social sciences ☐ Ecological, evolutionary & environmental sciences

For a reference copy of the document with all sections, see nature.com/documents/nr-reporting-summary-flat.pdf

Life sciences study design

All studies must disclose on these points even when the disclosure is negative.

Sample size	A formal sample size analysis was not performed as prior high-dimensional mass cytometry analyses GC effect on the surgical immune response was not available to anchor the power analysis. Instead, sample size was estimated based on previous data documenting the activation of STAT3 signaling pathways in CD4+T cells (Gaudilliere et al. Science Translational Medicine 2014), showing that 23 patients in each group would provided 90% power at p<0.05 to detect an intervention-related change in STAT3 phosphorylation in CD4+Tcells of 30%.
Data exclusions	Out of 63 patients enrolled in the study, samples for mass cytometry analysis was available for 58 patients due to technical inability to process the samples or patient refusal to provide a blood sample. The analysis was performed on samples from 58 patients.
Replication	Replication of findings: While prospective replication of the findings in an independent cohort is beyond the scope of the study, the reproducibility of the findings is ensured by the use of a stringent cross-validation method in our random-forest analysis of the mass cytometry data. At each individual time point, the mass cytometry data was used in a repeated leave-group-out cross validation approach to predict the probability that each sample came from a patient in the MP group. At each iteration, half of the samples were used to train a random forest model and predictions were made on samples in the remaining half of the data. Patient sample availability precluded technical replicates and individual samples were ran and analyzed once.
Randomization	A random allocation sequence (1:1 allocation rate, no block randomization) was created, and numbered and sealed envelopes were prepared to determine which arm of the study each patient would fall into. On the day of surgery, the envelopes were opened by a nurse not involved in any other aspect of the study, and either a single dose of 125 mg of methylprednisolone (Solu-Medrol®; Pfizer, Ballerup, Denmark) (MP group) or a single dose of isotonic saline (control group) was prepared in a separate room.
Blinding	MP or saline placebo were prepared in masked syringes and administered by one of two blinded investigators immediately after completion of spinal anesthesia. Investigators were blinded for the high-dimensional analysis of the data (Fig.1-2) and analysis of clinical outcomes (Fig. 6). Investigators were not blinded for the subsequent univariate analysis of individual features (Fig 3-5).

Reporting for specific materials, systems and methods

We require information from authors about some types of materials, experimental systems and methods used in many studies. Here, indicate whether each material, system or method listed is relevant to your study. If you are not sure if a list item applies to your research, read the appropriate section before selecting a response.

Materials & experimental systems

n/a	Involved in the study	
☐	☒	Antibodies
☒	☐	Eukaryotic cell lines
☒	☐	Palaeontology
☒	☐	Animals and other organisms
☐	☒	Human research participants
☐	☒	Clinical data

Methods

n/a	Involved in the study	
☒	☐	ChIP-seq
☐	☒	Flow cytometry
☒	☐	MRI-based neuroimaging

Antibodies

Antibodies used	Antibody reagents clones, concentrations, and providers are listed in Supplementary table 1.
Validation	Reproducibility of mass cytometry data: All antibody reagents utilized in the study were titrated to ensure specificity and reproducibility of the findings. For phenotypic marker antibodies, positive and negative cell populations are used to determine optimal antibody concentration and the sensitivity and specificity of the antibody signal. For positive controls in the validation of signaling antibodies, we use whole blood stimulated with LPS (expected positive signal for pERK1/2, pP38, pMK2, pCREB, pNF-κB and IκB degradation in TLR4-expressing innate immune cell subsets, such as classical monocytes, cMCs), or Interferon alpha (expected positive signal for pSTAT1, 3, 5, 6 in innate and adaptive cells, such as cMCs and CD4+ T cells). Negative control for signaling antibodies are the respective signal measured in the unstimulated blood sample.

Human research participants

Policy information about studies involving human research participants

Population characteristics	Inclusion criteria were: age 55 - 80 years and the ability to speak and understand Danish. Exclusion criteria were: general anesthesia, cancer, autoimmune diseases including rheumatoid arthritis, allergy or intolerance to methylprednisolone, local or systemic infection, continued systemic treatment with steroids within 30 days before surgery, insulin-dependent diabetes, atrial fibrillation, neurological diseases including Parkinson's, daily use of hypnotics or sedatives, alcohol use >35 units per week, active treatment of ulcers within 3 months before surgery, pregnancy, and breast-feeding or recent onset of menopause (<1 year) in women. Patient demographics and relevant clinical covariates are listed in Table 1: Patient and Procedural Characteristics<br><br>The manuscript was prepared according to the Consolidated Standards of Reporting Trials (CONSORT) recommendations for reporting randomized, controlled, clinical trials. The CONSORT chart is provided in Figure S1.
Recruitment	From February 2015 to December 2016, all patients undergoing elective, unilateral, primary Total Hip Arthroplasty at Copenhagen University Hospital, Bispebjerg and Frederiksberg, Denmark, were consecutively assessed for eligibility. After obtaining written informed consent, patients were enrolled before surgery, at their presurgical hospital visit.
Ethics oversight	This double-blind, placebo-controlled, randomized, and prospective clinical study was approved by the Danish Health and Medicine Authority (EudraCT 2015-000102-19), the Ethics Committee for the Capital Region of Denmark (H-15007653, protocol approval July 2015), the Danish Data Protection Agency.

Note that full information on the approval of the study protocol must also be provided in the manuscript.

Clinical data

Policy information about clinical studies

All manuscripts should comply with the ICMJE guidelines for publication of clinical research and a completed CONSORT checklist must be included with all submissions.

Clinical trial registration	The study was registered at ClinicalTrials.gov (NCT02542592).
Study protocol	The study protocol for the clinical trial has previously been published (https://www.ncbi.nlm.nih.gov/pubmed/29573263) and can be accessed upon request to Henrik Kehlet (Henrik.Kehlet@regionh.dk) or Dr. Viktoria Oline Lindberg-Larsen (viktoria_oline@hotmail.com)
Data collection	From February 2015 to December 2016, peripheral blood samples and clinical data were collected in enrolled patients at Copenhagen University Hospital, Bispebjerg and Frederiksberg, Denmark,
Outcomes	Our primary outcomes were peripheral immune cell distribution and intracellular signaling responses assessed using our mass cytometry immuno-assay.<br>Our secondary outcomes was to examine whether single-dose administration of GCs would improve patient-centered recovery outcomes, including pain and function, which were previously predicted by specific immune response patterns after surgical injury. Assessments were made 1 hour before and 1, 2, 7, 14 and 28 days after surgery as previously described in detail. Briefly, fatigue and resulting functional impairment were captured with the Surgical Recovery Scale (SRS; 17-100 = worst/best score), a well validated questionnaire specifically designed for the surgical setting. Pain and functional impairment of the hip were assessed with the Western Ontario and McMaster Universities Arthritis Index (WOMAC) adapted to the surgical setting. Pain scores range from 0-40 (no/worst pain), and function scores range from 0-60 (no/most severe impairment).

Flow Cytometry

Plots

Confirm that:

- [x] The axis labels state the marker and fluorochrome used (e.g. CD4-FITC).
- [x] The axis scales are clearly visible. Include numbers along axes only for bottom left plot of group (a 'group' is an analysis of identical markers).
- [x] All plots are contour plots with outliers or pseudocolor plots.
- [x] A numerical value for number of cells or percentage (with statistics) is provided.

Methodology

Sample preparation	Whole blood samples were collected in sodium-heparinized tubes at 6 time points (1 hour before surgery and 1, 6, 24, 48 hours, and 2 weeks after surgery). Within 30 minutes of phlebotomy, samples (1 mL) were processed and fixed in Smart Tubes (Smart Tube Inc., San Carlos, CA), and then immediately stored at -80°C. All samples were shipped on dry ice as a single batch to Stanford University (Stanford, CA) for further processing and analysis.<br><br>After thawing and erythrocyte lysis, samples were barcoded and stained with surface and intracellular antibodies using standardized protocols as previously described. In order to minimize experimental variability, samples corresponding to an entire time series were barcoded, stained, and run simultaneously on the mass cytometry instrument. In order to maximize the

sensitivity of the assay for detection of differences between the MP and control groups, sample time series from patients in the MP group were randomly paired with samples from patients in the control group, and paired sample time series were barcoded and run using the same barcode plate. Barcoded samples were analyzed at a flow rate of ~600-800 cells/s. The output FCS files were normalized and de-barcoded using MatLab-based software, as previously described. The resulting FCS files were uploaded to the Cell Engine (https://cellengine.com, Primity Bio, Fremont, CA) flow cytometry analysis platform.

Instrument	Samples were ran on a Helios mass cytometer instrument (Fluidigm, CA)
Software	Mass cytometry FCS files were normalized and debarcoded using Matlab based software available at https://github.com/nolanlab/single-cell-debarcoder/wiki/ The resulting FCS files were uploaded to the Cell Engine (https://cellengine.com, Primity Bio, Fremont, CA) flow cytometry analysis platform.
Cell population abundance	NA. Cell sorting was not utilized in our study.
Gating strategy	Gating strategy is provided in Supplementary Figure 2.

☒ Tick this box to confirm that a figure exemplifying the gating strategy is provided in the Supplementary Information.
