# Supplementary material for: Preferential inhibition of adaptive immune system dynamics by glucocorticoids in patients after acute surgical trauma
Source: Nat Commun. 2020 Jul 27;11:3737. doi: 10.1038/s41467-020-17565-y (PMC7385146; doi:10.1038/s41467-020-17565-y)
Supplement: Supplementary file 4 — Description of Additional Supplementary Files [file 41467_2020_17565_MOESM4_ESM.pdf]

Description of Additional Supplementary Files

Supplementary Movie 1. A non-linear dimensional reduction algorithm (Isomap) depicting all patients' immunological trajectories after surgery along the innate (X) and adaptive (Y) axes (MP in red, control in black). Isomap of immune trajectories can be found at: https://drive.google.com/file/d/1MsFM680OHR2aaSi44qekc_nFvEC-1OEQ/view
